# The RNA-binding protein FUS/TLS interacts with SPO11 and PRDM9 and localize at meiotic recombination hotspots

**DOI:** 10.1007/s00018-023-04744-5

**Published:** 2023-03-26

**Authors:** Teresa Giannattasio, Erika Testa, Ramona Palombo, Lidia Chellini, Flavia Franceschini, Álvaro Crevenna, Petko M. Petkov, Maria Paola Paronetto, Marco Barchi

**Affiliations:** 1 University of Rome “Tor Vergata”, Section of Anatomy, Via Montpellier, 1, 00133 Rome, Italy; 2 Laboratory of Molecular and Cellular Neurobiology, Fondazione Santa Lucia, CERC, 00143 Rome, Italy; 3 European Molecular Biology Laboratory, Neurobiology and Epigenetics Unit, Monterotondo, Italy; 4 The Jackson Laboratory, Bar Harbor, ME 04609 USA; 5 Department of Movement, Human and Health Sciences, University of Rome Foro Italico, Piazza Lauro de Bosis 6, 00135 Rome, Italy

**Keywords:** Meiosis, REC114, XY, H3K4me3, EWSR1, SPO11β, SPO11α

## Abstract

In mammals, meiotic recombination is initiated by the introduction of DNA double strand breaks (DSBs) into narrow segments of the genome, defined as hotspots, which is carried out by the SPO11/TOPOVIBL complex. A major player in the specification of hotspots is PRDM9, a histone methyltransferase that, following sequence-specific DNA binding, generates trimethylation on lysine 4 (H3K4me3) and lysine 36 (H3K36me3) of histone H3, thus defining the hotspots. PRDM9 activity is key to successful meiosis, since in its absence DSBs are redirected to functional sites and synapsis between homologous chromosomes fails. One protein factor recently implicated in guiding PRDM9 activity at hotspots is EWS, a member of the FET family of proteins that also includes TAF15 and FUS/TLS. Here, we demonstrate that FUS/TLS partially colocalizes with PRDM9 on the meiotic chromosome axes, marked by the synaptonemal complex component SYCP3, and physically interacts with PRDM9. Furthermore, we show that FUS/TLS also interacts with REC114, one of the axis-bound SPO11-auxiliary factors essential for DSB formation. This finding suggests that FUS/TLS is a component of the protein complex that promotes the initiation of meiotic recombination. Accordingly, we document that FUS/TLS coimmunoprecipitates with SPO11 in vitro and in vivo. The interaction occurs with both SPO11β and SPO11α splice isoforms, which are believed to play distinct functions in the formation of DSBs in autosomes and male sex chromosomes, respectively. Finally, using chromatin immunoprecipitation experiments, we show that FUS/TLS is localized at H3K4me3-marked hotspots in autosomes and in the pseudo-autosomal region, the site of genetic exchange between the XY chromosomes.

**Supplementary Information:**

The online version contains supplementary material available at 10.1007/s00018-023-04744-5.

## Introduction

The protein FUS (fused in sarcoma)/TLS (translocated in liposarcoma), along with the gene products encoded by the Ewing’s sarcoma breakpoint region 1 (*Ewsr1*) and the TATA-box binding associated factor 15 (*Taf15*), are RNA and DNA-binding proteins which belong to the FET (FUS, EWS, TAF15) family of proteins [1]. FET proteins mainly localize into the cell nucleus [2]. They are highly conserved and ubiquitously expressed and contribute to several basic biological processes in RNA and DNA metabolism, including the control of transcription, RNA processing and cytoplasmic fates of messengers RNAs [3–9], and detection of DNA damage [10–15]. They contain several conserved domains: a serine-tyrosine-glycine-glutamine (SYGQ) domain embedded in the DNA activation domain (AD), 3 glycine-arginine (RGG) rich regions that affect RNA binding, one conserved RNA-binding domain (RBD, formed by an RNA-recognition motif, RRM), and a zinc finger domain that is also involved in nucleic acid binding [1]. Interestingly FET proteins are also expressed in testis, where, in addition to controlling transcription of post-meiotic genes [16], they play key functions in the early stages of meiosis, as demonstrated by massive germ cell apoptosis in spermatocytes carrying ablation of either *Ewsr1* or *Fus* genes [17, 18].

In mammals, spermatogenesis starts within the seminiferous tubules, at postnatal age. In mice, a few days after birth, gonocytes differentiate into spermatogonia, which divide and subsequently differentiate into preleptotene spermatocytes 8–9 days post-partum (dpp). Next, as preleptotene cells enter meiosis, germ cell subpopulations appear consecutively and continuously, and seminiferous tubule becomes enriched of germ cell subpopulations at successive stages of differentiation. Thus, while prepuberal 9 dpp testes contain somatic cells and germ cells at early stages of differentiation (i.e., spermatogonia and preleptonema cells), seminiferous tubules of 10 dpp mice are additionally populated by prophase I cells at leptonema and zygonema, while early to late pachytene stage cells appear by 12–14 dpp. Subsequently, meiotic prophase I and II are completed and at adult age, post-meiotic spermatids and testicular sperm become the predominant cell types in the seminiferous tubules. The overall differentiation process from spermatogonia to mature spermatozoa requires ~ 35 days, while successive waves of spermatogenesis begin approximately once every 8 to 9 days [19]. It follows that while during the first wave of spermatogenesis meiotic cells appear in seminiferous tubules semi-synchronously, in the adult testes successive waves overlap each other, resulting in the association of cells that characterize the stages of spermatogenesis [20, 21]. During the prophase of meiosis I, homologous chromosomes (henceforth referred to as homologs) from differing parental origins (each consisting of two sister chromatids), pair, and synapse. Following the formation of physical links (chiasmata), they subsequently align and move to opposite poles at metaphase I. At the second meiotic division, sister chromatids separate to form round haploid spermatids, which elongate and eventually mature into spermatozoa. In normal cells, pairing between the homologous chromosomes (that is, approaching and juxtaposing of the chromosomes) begins premeiotically with recombination-independent mechanisms [22, 23]. However, stable pairing maintenance and synapsis requires recombination [22], which is initiated by the formation of double-stranded DNA breaks (DSBs) by the SPO11/TOPOVIBL complex [24–29]. After their formation, the DSB ends are repaired by homologous recombination, and homologous synapsis is stabilized by the formation of a zipper-like proteinaceous structure called the synaptonemal complex (SC) [30]. Recombination ultimately gives rise to both crossover (CO) and non-crossover (NCO) products. NCOs form with a much higher frequency than COs, and the formation of intermediates of NCOs promotes homologous pairing. Only one to two COs form in each chromosome in mice, leading to the formation of interhomolog DNA links cytologically identifiable as chiasmata [24, 31, 32]. Proper formation of meiotic DSBs requires not only the SPO11/TOPOVIBL complex, but also the expression of SPO11 auxiliary factors, including IHO1, MEI1, MEI4, and REC114 [33–40]. These proteins assemble along the chromosome axes prior to DSB formation and form cytologically visible foci that are essential for activating SPO11/TOPOVIBL function. When the expression of one of these protein factors is impaired, DSB formation fails, with the consequent failure of homologs synapsis [33–40]. Proper establishing of synapsis between homologs requires that DSBs are initiated at multiple sites along the length of chromosomes. For this reason, the DSB numbers mediated by SPO11/TOPOVIBL largely exceed that of crossovers. Accordingly, if the number of DSBs is reduced below a critical threshold, homolog synapsis fails, with the consequent elimination of defective spermatocytes by apoptosis [41, 42]. In addition to a numerical constraint, it is necessary that DSBs are made in specific genomic regions called hotspots. A widely conserved methyltransferase responsible for marking DSBs hotspot sites in mammals is the meiosis-specific protein PR domain-containing 9 (PRDM9) [43]. After its interaction with the chromatin remodeler HELLS [44, 45], PRDM9 binds onto DNA, and trimethylates histone 3 at Lys-4 (H3K4me3) and Lys-36 (H3K36me3) allowing access onto the DNA of SPO11/TOPOVIBL [46–50]. In mice displaying inactivation of the *Prdm9* gene, DSBs form in normal numbers, but occur in functional regions, such as promoters and enhancers, which are rarely targeted in wild-type mice [47, 51]. This parallels a defect in the repair of DSBs, with the consequent failure of synapsis between the homologs and cell death [20, 47, 51, 52]. It is noteworthy that the phenotype of mice with functional inactivation of *Prdm9* resembles that of *Ewsr1* and *Fus/Tls* knockout mice, in which DSBs form in normal numbers, but homolog synapsis fails, causing male sterility [17, 18, 53]. In an effort to understand the specific role of EWS in meiosis, it was recently shown that EWS is a direct binding partner of PRDM9 [54], and that its genetic ablation in spermatocytes leads to a reduced level of H3K4me3 and H3K36me3 at hotspots, accompanied by the disappearance of a selection of H3K4me3 hotspots and the shift of a fraction of DSB hotspots to heterochromatin-rich regions, normally cold in wild-type cells. This suggests that *Ewsr1* supports proper PRDM9 and DSB activity in spermatocytes [53].

Formation of DSBs occurs in the context of the spatial organization of meiotic chromosomes, which form chromatin loops that extend from a linear protein axis. Based on the yeast model, it is thought that the DSB machinery (assembled on axes) captures and breaks loop DNA [55, 56]. Given that EWS and PRDM9 interact in vitro and in vivo and that EWS interacts with the chromosome axis bound cohesin REC8 [53, 54], it has been proposed that EWS participates in the association of H3K4me3-marked hotspots with the axis [53], thus marking the site of DSB formation and genetic recombination [57–59]. However, since a significant portion of PRDM9-dependent hotspots is still forming DSBs in *Ewsr1* deleted spermatocytes [53], EWS is not likely the only protein directing PRDM9-marked DNA to the axis. Given the meiotic phenotype of *Fus/Tls* knockout mice [18] and the high level of homology between FUS/TLS (henceforth called FUS) and EWS, we asked whether FUS could also play a regulative function in DSB formation. A comparison of *Ewsr1*^*−/−*^ and *Fus*^*−/−*^ phenotypes in spermatocytes revealed that synaptic defects of *Fus*^−/−^ spermatocytes are more severe than in *Ewsr1*^*−/−*^ cells [17, 18]. This suggests that the two proteins might perform similar but not overlapping functions during meiosis.

Here, we demonstrate that FUS interacts with PRDM9 and SPO11 in vitro and in vivo. Furthermore, we show that FUS interacts with REC114, one of the axis-bound proteins required to activate the SPO11/TOPOVIBL function. Finally, we prove that FUS localizes onto chromatin at H3K4me3 hotspot sites of the autosomes, and in the pseudo autosomal region (PAR), the site of genetic exchange between the XY chromosomes.

## Results

### FUS expression is finely regulated during male meiosis

**Fig. 1:**
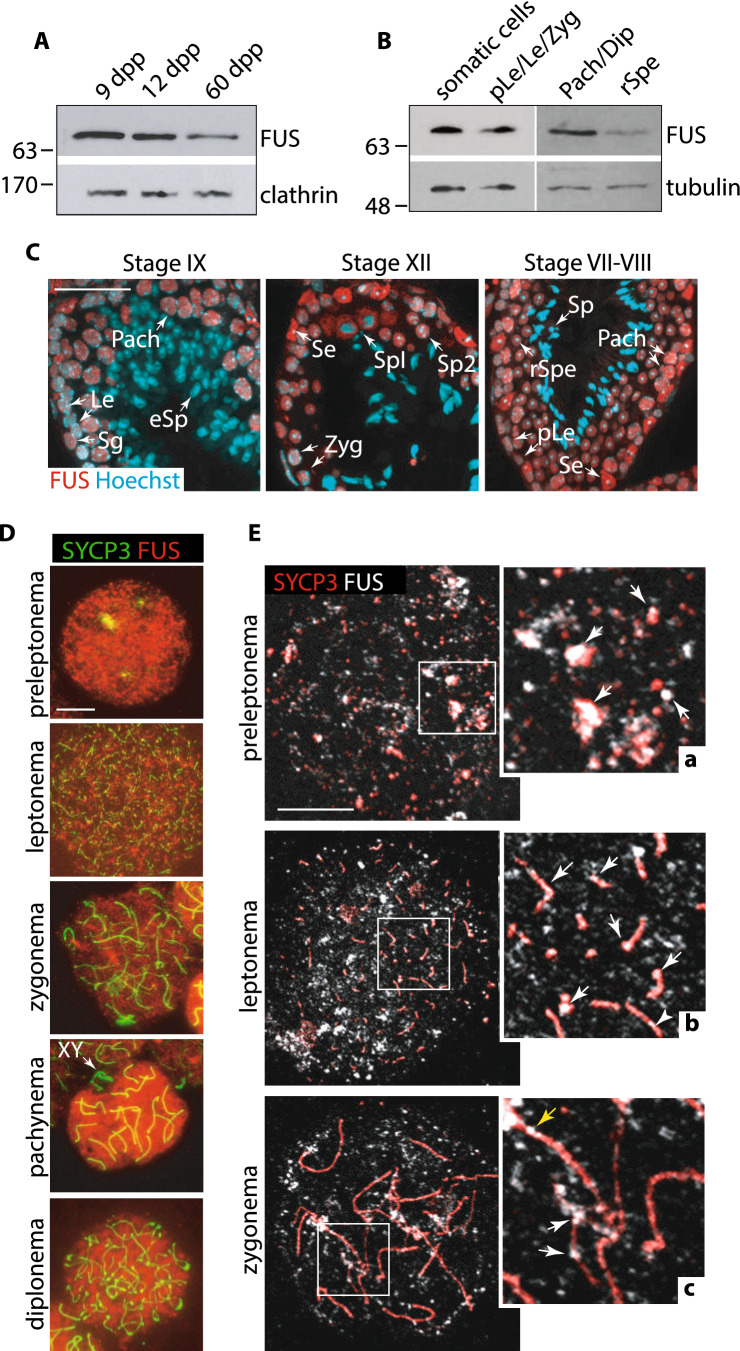
Analysis of FUS expression and localization. **A** Western Blot (WB) of FUS expression in total testes extracts from mice at the indicated age. At least two testes were used for each time point. The expression of clathrin proteins served as a normalizer. **B** WB analysis of FUS protein level in enriched cell populations of the testis. Somatic cells and pre-leptotene (pLe)/leptotene (Le)/zygotene (Zyg) stage cells were isolated from five wild-type 10 dpp old mice; pachytene/diplotene (Pach/Dip) stage cells and round spermatids (rSpe) were isolated from four wild-type adult mice. Tubulin served as a normalizer. **C** Immunolocalization of FUS (red) and cell nuclei (blue) in seminiferous tubules at the indicated stages of the epithelial cell cycle. The white arrows point to somatic Sertoli cells (Se) and germ cells at different stages of development: spermatogonia (Sg), primary spermatocytes (SpI), secondary spermatocytes (SpII), elongated spermatids (eSp), and spermatozoa (Sp). Magnification bar represents 50 μm. **D** Immunolocalization of FUS (red) and SYCP3 (green) on surface chromosome spreads of germ cells, at the indicated stages of development. Images were captured using an inverted fluorescence microscope. Magnification bar represents 10 μm. **E** Confocal images of FUS (gray) and SYCP3 (red) on surface chromosome spreads at the indicated stages. The white arrows in magnifications (a–c) point at sites of colocalization of FUS with the unsynapsed chromosome axes identified by SYCP3. The yellow arrow in **C** points to FUS on synapsed chromosome axes. Magnification bar represents 10 μm

Early studies have demonstrated that FUS is abundantly expressed in mouse pachytene spermatocytes, whereas its expression was weakened in round spermatids [18]. To better characterize the global expression of FUS in mouse testes, we collected whole testes at different time points from prepuberal to adulthood and performed a Western blot analysis. As shown in Fig. 1A, FUS expression was high in the 9 dpp and 12 dpp testes, which are enriched in somatic/premeiotic and early pachytene stage cells, respectively; while it decreased in the adult testes, suggesting elevated expression in somatic cells and prophase I germ cells, and low expression at later stages of meiosis. To confirm this observation, we performed a protein expression analysis from partially purified testis cell populations. Somatic cells and a mixed population of preleptotene/leptotene and zygotene stage cells were isolated from 10 dpp mice, according to the protocol described by Rossi et al. [60]. Isolated populations of germ cells at pachynema/diplonema and round spermatids were obtained from adult mice by centrifugal elutriation [61]. As shown in Fig. 1B, we found that FUS is expressed at a high level in somatic cells and in prophase I stages up to pachynema/diplonema, and weakly expressed in round spermatids. Next, to localize FUS in specific germ cell subpopulations, we immunolocalized it on testis sections. Cell types were identified according to the nuclei morphology and germ cell composition of the seminiferous tubules at the indicated stages of the epithelial cell cycle, as previously described [21, 62]. As shown in Fig. 1C, FUS was highly expressed in somatic Sertoli cells and was present at a reduced level in germ cells from leptonema to secondary spermatocytes. The expression further declined in round spermatids, and it was undetectable in elongated spermatids and spermatozoa. Next, to understand whether FUS was associated with chromatin in germ cells, surface-spread chromosomes from juvenile wild-type mice were prepared. To identify germ cell substages, staining with the SC component SYCP3 was performed, which allows precise identification of prophase I cells [63, 64]. We found that FUS associates with chromatin and appeared to be distributed throughout the nucleus area from preleptonema to diplonema but was excluded at pachynema from the area of X and Y chromatin, known to undergo transcriptional silencing (Fig. 1D) [18, 65], confirming previous observations [18]. By carefully analyzing the localization pattern of FUS, we observed some areas of co-occurrence of SYCP3 and FUS signals at zygonema, (Fig. S1A), however, whether this occurred more frequently than randomly was difficult to determine, because of the widespread pattern of FUS. To further evaluate this aspect, we resorted to confocal microscopy, which allowed for reduced FUS signal noise. We observed that the protein appeared to colocalize partially with the nascent SC stretches at preleptonema and leptonema (see magnifications in Fig. 1E a, b). This was confirmed by colocalization analysis of fluorescent signals using Pearson's colocalization coefficient (PCC). Rotation of FUS channels of 180 degrees served as a control for random colocalization (PCC 0.3 ± 0.017; Vs Ctr 0.05 ± 0.02, Fig. S1 B–D). At mid to late zygonema FUS remained partially associated with unsynapsed and synapsed chromosome axes as scattered foci (Fig. 1E c and S1E) persisting as spotted and weak more continuous staining on the axes, at pachynema (Fig. S1F).

### FUS interacts with PRDM9 regardless of PRDM9 methyltransferase activity

**Fig. 2:**
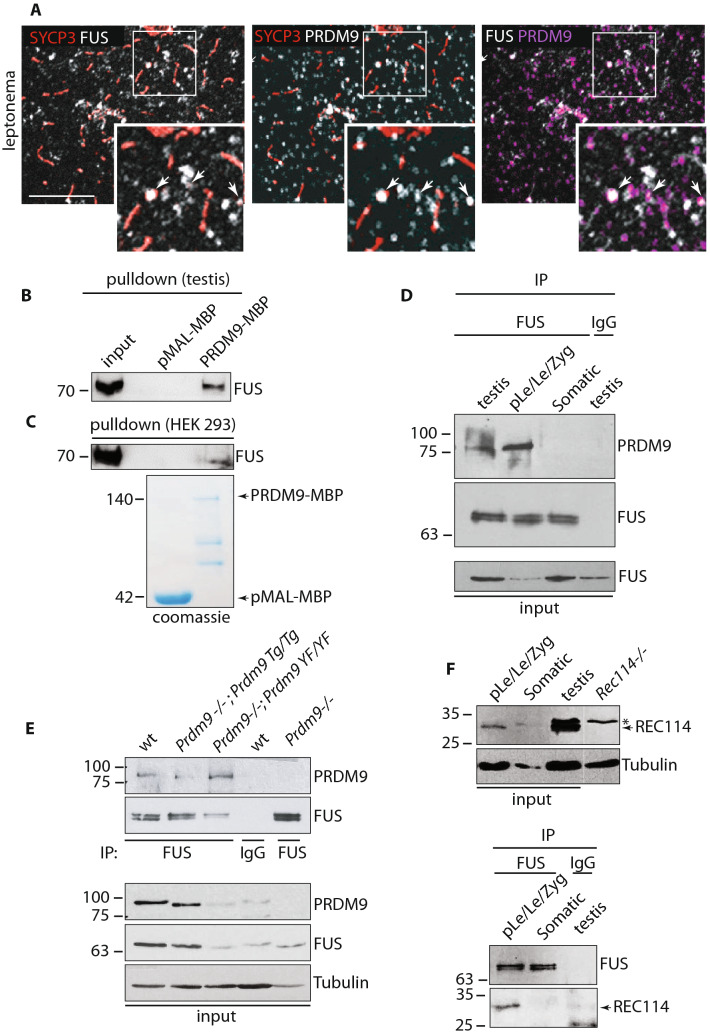
FUS colocalizes/interacts with PRDM9 and coimmunoprecipitates with REC114. **A** Confocal imaging of FUS, SYCP3, and PRDM9 in surface chromosome spreads at leptonema. The white arrows indicate SYCP3 colocalization with FUS (left panel), SYCP3 colocalization with PRDM9 (mid panel) or FUS colocalization with PRDM9 (right panel). Magnification bar represents 10 μm. **B** Pulldown by maltose-binding protein (pMAL-MBP) and PRDM9-maltose binding protein (PRDM9-MBP) of FUS from 11–12 dpp total testis extracts (four testes per lane). **C** Pulldown by the PRDM9-MBP protein of endogenously expressed FUS in HEK293 cells. The Coomassie stained gel shows the purified PRDM9-MBP protein. **D** IP/WB analysis of FUS and WB of PRDM9 in total extracts from juvenile mice testes, isolated populations of germ cells and somatic cells obtained from 10 dpp old mice. IP from somatic cells of the testes and 10 dpp testes with anti-FUS and nonspecific IgG, respectively, served as negative controls. pLe/Le/Zyg is a mix population of pre-leptotene, leptotene and zygotene stage cells. **E** IP/WB analysis of FUS and WB of PRDM9 from total testis extracts from mice of the indicated genotypes. Wild types (wt) are six testes from 14 dpp old C57BL6/J mice, *Prdm9*^*−/−*^; *Prdm9*^*Tg/Tg*^ are two testes from adult mice, *Prdm9*^*−/−*^; *Prdm9*^*YF/YF*^ are four testes from adult mice, *Prdm9*^*−/−*^ are two testes from adult mice. **F** Top panel, identification of the REC114 specific band in inputs used for FUS immunoprecipitation. *The Rec114*^*−/−*^ adult testes served as a control. pLe/Le/Zyg is a mix population of pre-leptotene, leptotene and zygotene stage cells obtained from 10 dpp old mice. “Somatic” indicates a mix population of testicular somatic cells obtained from 10 dpp old mice. The total testis extract was prepared from 14 dpp old mice. Lower panel, IP/WB analysis of the FUS-REC114 interaction in the extracts indicated above. The IP with a non-specific IgG served as negative control. Seven testes were used for each point. The asterisk indicates a non-specific band

Recent studies demonstrated that the FET family member EWS interacts with PRDM9 through its functional zinc-finger Ranbp2-type domain, which is conserved among the FET family [1, 54]. PRDM9 is expressed from pre-leptonema to zygonema stages [52] (Fig. S2A) and a proportion of the protein is expected to localize on meiotic chromosome axes [54], where it is believed to play a function in directing SPO11-TOPOVIBL activity at hotspot sites [47, 48, 66]. To understand whether FUS and the axis bound proportion of PRDM9 colocalize onto chromosome axes, we immunolocalized FUS and PRDM9 on spermatocytes. As shown in Fig. 2A, a fraction of PRDM9 localizes on SYCP3 positive chromosome axes (SYCP3/PRDM9 PCC at preleptonema/leptonema is 0.17 ± 0.02; Vs Ctr 0.02 ± 0.006, Fig. S2B) and FUS/PRDM9 signals partially overlap (FUS/PRDM9 PCC at preleptonema/leptonema is 0.3 ± 0.03; Vs Ctr 0.05 ± 0.01, Fig. S2C), suggesting a possible interaction. To further investigate it, we performed a FUS pull-down using PRDM9 as bait. To this end, a full-length *Prdm9*^*Dom2*^ allele expressed in *E. coli* with a C-terminal maltose-binding protein (MBP) tag (PRDM9-MBP) was purified and incubated with total extracts of juvenile mouse testes (Fig. 2B) or human embryonic kidney 293 (HEK-293) cells, which endogenously expresses FUS (Fig. 2C). We found that FUS and PRDM9 interact in vitro under both conditions. Next, to test the interaction of proteins in vivo, we immunoprecipitated FUS from mouse testes extracts. Since PRDM9 and FUS are coexpressed at early meiotic stages (Fig. 1D and S2A), we immunoprecipitated FUS from juvenile (12 dpp) mouse testis extracts, which are enriched in germ cells from preleptotene to early pachytene stages (Fig. S2D) and an enriched fraction of cells at preleptonema/leptonema/zygonema. As a control for PRDM9 signal specificity we performed a side-by-side immunoprecipitation of FUS from somatic cells of the testis, which lacks PRDM9 [52]. The interaction between PRDM9 and FUS was readily visible in immunoprecipitates from juvenile testes and even more in those cell extracts from preleptonema/leptonema/zygonema cells (Fig. 2D). This demonstrates that FUS and PRDM9 also interact in vivo. Next, to understand whether the establishment of a physical interaction between FUS and PRDM9 can also occur before the epigenetic modifications introduced by PRDM9, we analyzed the interaction in testicular extracts of *Prdm9*^*−/−*^ transgenic mice in which the function of *Prdm9* was complemented by a transgene (Tg) that expresses a functional *Prdm9* (i.e. *Prdm9*^*−/−*^; *Prdm9*^*Tg/Tg*^ genotype), or a *Prdm9* that contains the Y/F inactivating substitution mutation in the catalytic domain (i.e. *Prdm9*^*−/−*^; Tg*Prdm9*^*YF/YF*^) [47]. We observed that FUS coimmunoprecipitates with PRDM9 carrying the Y/F substitution, demonstrating that the interaction does not need PRDM9 catalytic function (Fig. 2E), and thus it may possibly occur before of H3K4me3 and H3K36me3 modifications of the chromatin. Importantly, all pull-down and immunoprecipitations in this study were performed using extracts pre-incubated with benzonase, which digests nucleic acid. Thus, the observed interactions occur through a (direct or indirect) protein–protein binding.

### FUS interacts with SPO11 and REC114 during the early prophase I

**Fig. 3:**
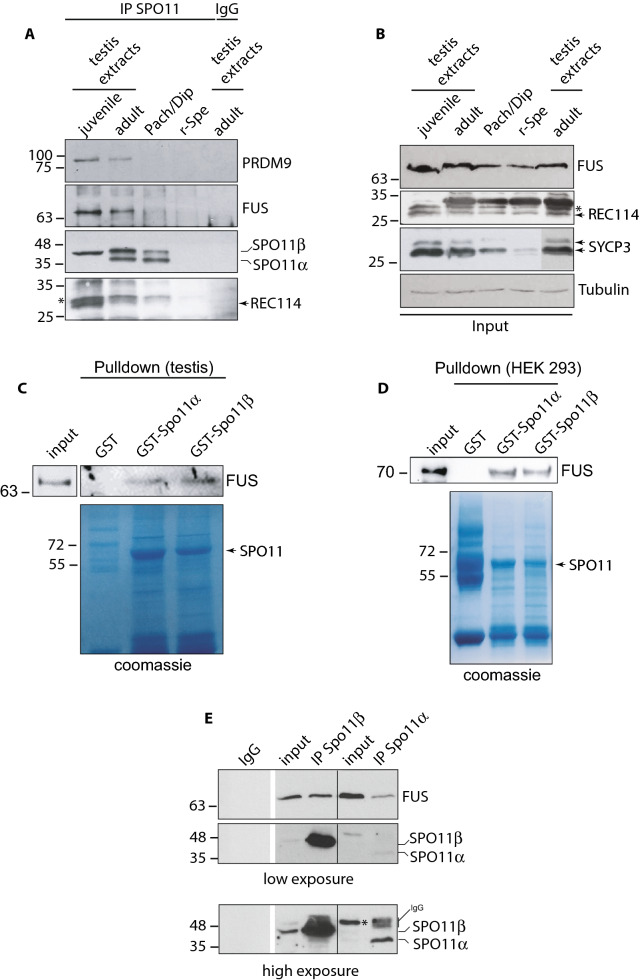
FUS interacts with SPO11 in vivo and in vitro. **A** IP/WB analysis of SPO11 and WB of FUS, PRDM9, and REC114 coimmunoprecipitating from juveniles and adult mice total testes extracts and from isolated populations of germ cells. Pach/Dip and rSpe are pachytene/diplotene stages enriched fractions and round spermatids, respectively, obtained from adult mice. Total extracts of juvenile mice testes were prepared from five 12 dpp mice. The total extracts of the testes of adult mice were from a 6-month-old mouse. The IP with a non-specific mouse IgG served as negative control. The immunoprecipitation of SPO11 from a purified fraction of r-Spe served as a control for PRDM9 and REC114 signal specificity. The asterisks indicate nonspecific bands (see Fig. 2F, top panel). **B** FUS and REC114 expression in total extracts of the input. SYCP3 served as a measure of spermatocyte cell content, while tubulin was used as an independent loading control. **C** Pulldown by the SPO11β-GST and SPO11α-GST recombinant proteins, of FUS from 12 dpp testis extracts, or **D** from extracts of HEK293 cells. Coomassie-stained gels in **B** and **C** show SPO11-GST fusion proteins. **E** IP/WB analysis of SPO11β/SPO11α and WB of FUS coimmunoprecipitated from total testis extracts of adult knock-in mice expressing only either SPO11β or SPO11α cDNA. The higher exposure of the film of SPO11 IP is shown to allow a better visualization of the SPO11α splice isoform. IgG indicates immunoglobulin. The asterisk in the input indicates a nonspecific band

Seminal studies in *Saccharomyces cerevisiae* proved that hotspots are localized in chromatin loops, while DSB formation occurs on the chromosome axis [55, 57, 58] after functional activation of SPO11 by the complex of axis-bound proteins that includes REC114 [33–40, 55, 58, 67, 68]. As FUS was found to be partially localized on the chromosomal axes, we asked whether it could interact with REC114. First, to analyze the expression pattern of REC114, we collected an enriched-fraction of cells at preleptonema/leptonema/zygonema from 10 dpp mice and compared it with that of aged-matched somatic cells of the testis and total testis. As shown in Fig. 2F (top panel), we observed that while in the total testis extract the antibody detected a doublet, in that from cells at the preleptotene/leptotene/zygotene stages it recognized only the lower band, while only the upper band was detected in the extract of somatic cells. From comparison with the total testis extract from *Rec114*^*−/−*^ mice we established that the lower band of the doublet was specific, confirming previous observations [37] obtained with the antibody in our supply. Next, we immunoprecipitated FUS and examined the presence of REC114 in the immunoprecipitate. We observed that REC114 was coimmunoprecipitated with FUS in the preleptonema/leptonema/zygonema enriched fraction, while no interaction was observed in somatic cells, as well as in testes extract subjected to immunoprecipitation with a nonspecific IgG (Fig. 2F, lower panel). We concluded that FUS interacts with REC114 in vivo. Next, since REC114 is a direct binding partner of TOPOVIBL [69] and the latter complexes with SPO11 [26], we asked whether FUS might be part of the protein complex containing SPO11. Therefore, we immunoprecipitated SPO11 from juvenile and adult testicular extracts and analyzed its interaction with FUS. As shown in Fig. 3A, we observed a strong interaction between FUS and SPO11 in extracts from juvenile testes and a weaker interaction in adults. FUS was not detected when SPO11 was immunoprecipitated from an enriched fraction of cells at the pachytene/diplotene stages, despite comparable levels of FUS input (Fig. 3B) and SPO11 expression (Fig. 3A). This suggests that coimmunoprecipitation occurs in germ cells at the early stage of prophase. Furthermore, we found that PRDM9 coimmunoprecipitated strongly with SPO11 in extracts of juvenile testes, while, in agreement with the observation that PRDM9 expression is restricted to cells of early meiotic stage, the interaction was weaker in testes extracts from adult mice and absent in germ cells in stages beyond zygonema (Fig. 3A, top panel). Subsequent analysis of the presence of REC114 in the immunocomplex revealed that, despite the fact that REC114 was also detectable in total extracts of both pachynema/diplonema and round spermatids (see input in Fig. 3B), the protein clearly coimmunoprecipitated with SPO11, only in extracts from juvenile testes. Conversely, it was weakly detectable in extracts from adult testes and undetectable in fractions enriched with pachynema/diplonema and in rounds spermatids (Fig. 3A). These experiments let us conclude that the SPO11/FUS/PRDM9 and REC114 interactions occur in vivo in the early stages of prophase I. Next, given the strict structural homology of FUS and EWS, we tested whether EWS also coimmunoprecipitates with SPO11. We found EWS immunoprecipitated with SPO11 and FUS (Fig. S2E), suggesting that FUS and EWS may have, at least, partially redundant functions (see discussion). Moreover, we observed that FUS and PRDM9 also coimmunoprecipitate in the testes of *Spo11*^*−/−*^ mice (Fig. S2F), indicating that they interact regardless of SPO11 and the formation of DSBs.

### FUS interacts with both SPO11β and SPO11α isoforms

In mice and humans, the *Spo11* gene produces two major splicing variants: *Spo11β* and *Spo11α* [70, 71], whose expression is regulated in a timely manner during meiosis. SPO11β is expressed earlier, when DSBs form nucleus-wide, whereas SPO11α start to be expressed in late prophase I. The latter, likely plays a function in DSB formation in the PAR, the site of genetic exchange between XY chromosomes [72]. Our immunoprecipitation experiments showed that FUS interacts with SPO11β (first lane in Fig. 3A). However, whether it is also capable to interact with SPO11α remained unknown, as in adult wild-type testis the latter is always expressed concomitantly with SPO11β. To answer this question, using juvenile testis extracts, we performed a GST-pull down assay by using SPO11α and SPO11β recombinant proteins (see Coomassie in Fig. 3C) as baits. We observed that FUS interacts in vitro with both SPO11 splice isoforms (Fig. 3C). Afterward, to understand whether the interaction requires the expression of testicular-specific factors, we repeated the pulldown using HEK293 cells as a source of FUS. Again, we observed the interaction of FUS with both SPO11β and SPO11α (Fig. 3D). Additionally, taking advantage of two mice models generated in our laboratory, expressing either SPO11β or SPO11α (Barchi M., unpublished data), we immunoprecipitated SPO11 and asked if the interaction of FUS with either of the splice isoforms also occurs in vivo. As shown in Fig. 3E, after immunoprecipitation of SPO11β from testes extract, we easily detected its interaction with FUS in vivo. Similarly, FUS was revealed in the immunoprecipitate from testes expressing only SPO11α, although the latter was expressed at a lower level than SPO11β (compare the two different exposures in Fig. 3E). We concluded that FUS interacts with both SPO11β and SPO11α splice isoforms, both in vitro and in vivo.

### FUS localizes onto chromatin at hotspot sites marked by H3K4me3

**Fig. 4:**
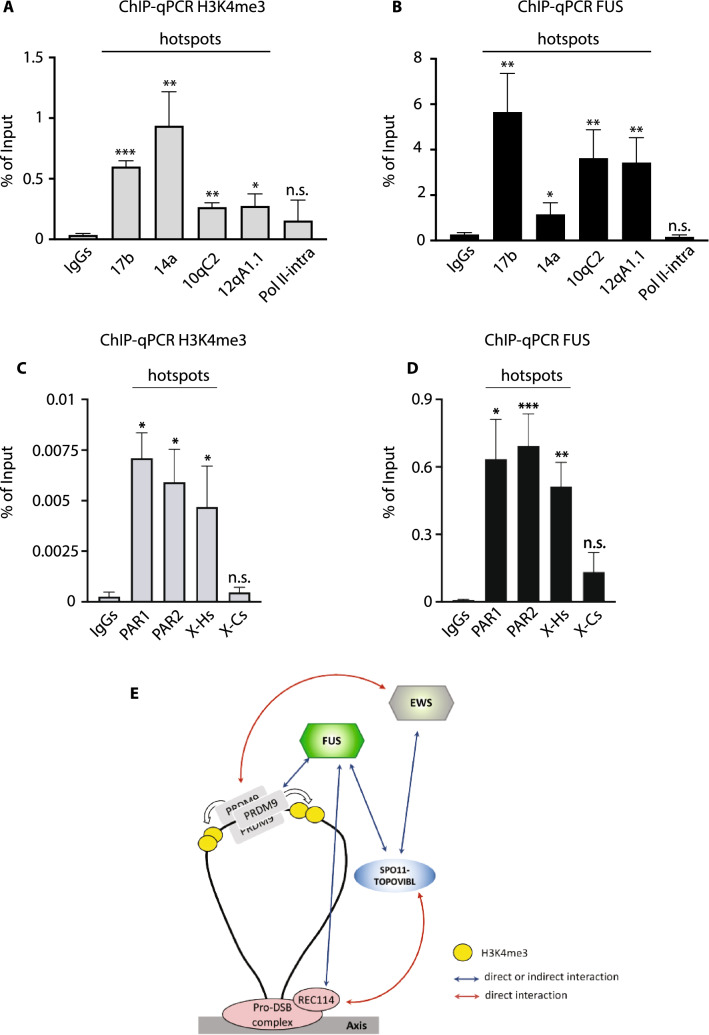
FUS localizes at H3K4me3-marked hotspots. **A** Chromatin Immunoprecipitation (ChIP) of H3K4me3 and quantitative Real-Time PCR (qPCR) of the indicated PRDM9^Dom2^ hotspots. The intragenic region of Pol II (Pol II-intra) is an H3K4me3 coldspot. **B** ChIP-qPCR of FUS at the indicated hotspots and the coldspot. **C** ChIP-qPCR of H3K4me3 and qPCR at two distinct pseudoautosomal regions named 1 and 2 (PAR1 and PAR2). X-Hs is a PRDM9^Dom2^ hotspot of a non-PAR genomic region of the X chromosome. X-Cs is a PRDM9^RJ2^ hotspot in a non-PAR genomic region of the X chromosome, expected to be a coldspot in the C57 background. **D** ChIP-qPCR of FUS at the indicated hotspots. In all cases, the analyses were performed from at least three 12dpp old mice testes, and the data were normalized against the input. Nonspecific IgG-based qPCR served as a control. Each ChIP-qPCR experiment was performed at least in triplicate. Error bars are mean ± standard error of the mean. Statistical significances (*p < 0.05; **p < 0.005; ***p < 0.001; n.s. = not significant) indicates enrichment to IgGs (two tails *t*-test). **E** Putative model of the action of FUS in early meiosis, according to the axis loop tethering model. FUS by interacting concomitantly (directly or indirectly) with PRDM9 and SPO11 provides a link between the H3K4me3-marked chromatin loops and the SPO11/TOPOVIBL cleavage complex. The interaction of FUS with REC114 may promote the association of the PRDM9-marked hotspot with the axis. EWS by interacting (directly) with PRDM9 and (directly or indirectly) with SPO11, may support the function of FUS

If the interaction of FUS with PRDM9, SPO11, and REC114 is of functional relevance to the formation of DSBs, we would expect FUS to localize at hotspot sites. Therefore, we performed a chromatin immunoprecipitation (ChIP) experiment at characterized H3K4me3 PRDM9 hotspots. In mice and humans, the binding of PRDM9 to genomic sites varies according to the array of its zinc finger domain. Different mouse strains express different *Prdm9* alleles, with consequent variations in the genome-wide distribution of recombination events [48, 51, 73]. To address the DNA binding ability of FUS at hotspots, we tested its enrichment at the hotspot sites identified by the *Prdm9*^*Dom2*^ allele from the C57BL6/J strain. We selected four sites, namely: 17b, 14a [47], 10qC2, and 12qA1.1 [51, 74]. First, to determine if the above are bona fide PRDM9-dependent hotspots, we looked at the enrichment of PRDM9, H3K4me3, H3K36me3, and DMC1 using a published dataset [47, 75]. As shown in Fig. S3, the selected regions are highly enriched of H3K4me3 and DMC1. An enrichment was also found for H3K36me3 and PRDM9, although the peaks are not all equally hot. We concluded that the genomic regions we selected are genuine hotspots. Next, we experimentally assessed the enrichment of H3K4me3 at these hotspots by ChIP from juvenile testes. As expected, we found that H3K4me3 was significantly enriched at all hotspot sites (Fig. 4A), while the intragenic region of Polr2a, which has been shown to be a cold spot for H3K4me3 (Fig. S3 and [74]), was used as a negative control. Next, we analyzed FUS enrichment at the same genomic locations. Remarkably, we observed enrichment of FUS on the chromatin of all hotspot sites, but not in the coldspot region (Fig. 4B). These experiments let us conclude that FUS binds onto chromatin at PRDM9-marked hotspot.

Since FUS also coimmunoprecipitates with SPO11α, we also asked whether FUS was also associated with the large H3K4me3 hotspot in the PAR [51]. Data mining in ChIP-seq genome-wide datasets [75] allowed the selection of two PAR regions (hereafter called PAR1 and PAR2) highly enriched in H3K4me3 and DMC1 reads (Fig. S3). H3K4me3 ChIP analysis of these regions confirmed that these are true hotspots (Fig. 4C). In the experiment, the non-PAR X- hotspot and the X-chromosome RJ2 strain hotspot were used as positive and negative controls, respectively. Notably, we found that FUS binds specifically at the non-PAR X-hotspot and PAR1 and PAR2 genomic regions (Fig. 4D).

## Discussion

PRDM9 plays a key function in meiotic recombination, as it specifies the hotspots, the sites of genetic recombination. By promoting the trimethylation of H3K4 and H3K36, PRDM9 modifies the nucleosomes near its binding site to DNA [46, 66], thus creating an environment conducive to the formation of DSBs by the heterotetrameric complex SPO11/TOPOVIBL [46, 58, 76]. DSBs form in conjunction with the development of axial chromosome structures, to which chromatin loops connect forming linear arrays. Studies in *S. Cerevisiae* indicate that DSBs mostly occur at sites not constitutively axis-associated but within loops that are tethered to the axis before or at the time of DSB formation through the action of SPO11, by accessory proteins that assemble onto the axis [55]. To date, it is unknown whether the loop/axis-tethering model applies to mammals. However, observations that mammals express homologous auxiliary factors to those of yeast, that these assemble as foci on the chromosome axis [34–40, 58] and that DSB markers associate with the axis [77], suggest that the mechanism may be similar. This allows for the possibility that the SPO11/TOPOVIBL complex may first bind at chromatin loops at sites specified by PRDM9, then it is tethered to the chromosome axis, where the SPO11 is activated by its interaction with the auxiliary factors.

In recent years, numerous efforts have been devoted to clarifying the molecular steps occurring from PRDM9 binding to DSB formation. By looking at PRDM9 interactors, it has been found that the RNA-binding protein EWS interacts with the N-terminal of PRDM9 in vitro through its Ranbp2-type zinc finger functional domain [54, 78], and that in vivo, EWS binds to REC8, a protein component of the linear core forming the chromosome axes, and with SYCP3, a component of the SC [54]. This allowed the authors to propose a role for EWS in the bridge of hotspot DNA from out-of-chromosome loops down to the chromosome axis [54]. Successively, using the *Ewsr1* conditional knockout mouse model, it was demonstrated that EWS is not strictly required for the designation of hotspot sites by PRDM9. Instead, it mostly plays a role in empowering the deposition of H3K4me3 and activation of SPO11 at the hotspots [53]. This predicts the presence of additional (*Ewsr1-*independent), or backup mechanisms, that guarantee the designation of PRDM9-dependent hotspots. Although protein factors involved in this mechanism are currently unknown, it is plausible to expect that failure of association between hotspot marks and SPO11 activity would cause a meiotic phenotype highly similar to that of *Prdm9*^*−/−*^ spermatocytes, showing normal DSB number and inefficient recognition and synapsis of homology throughout the nucleus, caused by the lack of hotspots designation [51, 52]. In this respect, our attention was drawn to the phenotype of *Fus*^*−/−*^ spermatocytes. Like *Prdm9*^*−/−*^ cells, spermatocytes lacking *Fus* show nucleus-wide mispaired synapsis of the homologs, besides efficient formation of DSBs [18]. Thus, given the structural similarities among FET proteins, we sought to ask whether FUS could play a role in the initiation of meiotic recombination. First, we asked whether FUS associates with the chromatin and chromosome axes. We observed a nucleus-wide distribution of FUS onto chromatin, as well as focal colocalization with the chromosome axe-associated protein SYCP3. Next, given the conservation of the Ranbp2-type zinc finger domain among FET proteins, we asked whether FUS colocalizes and interacts with PRDM9. We observed that a fraction of FUS and PRDM9 colocalize on the axes and that FUS and PRDM9 interact in vitro and in vivo in the absence of nucleic acids, specifically in early meiotic cells, the time of development when SPO11-mediated DSBs form physiologically [27]. The interaction between FUS and PRDM9 also occurred using HEK293 as a source of FUS, suggesting that it does not require the expression of testicular-specific factors and therefore may be direct. Given the well-known function of PRDM9 in the specification of the DSB sites, these results suggested a function of FUS at the DSB sites. It is interesting that immunoprecipitation of FUS from testis shows the presence of a doublet, which could be the result of post-translational modifications of the protein, or an as yet uncharacterized splicing isoform. According to the loop/axis-tethering model, activation of the SPO11 function is thought to occur on the chromosome axis where it interacts with axis-bound auxiliary factors, including REC114 [56, 58]. To understand whether FUS may interact spatially with one of these factors, we tested its interaction with REC114. We observed that the two proteins were coimmunoprecipitated, suggesting a putative function of FUS at the sites where the SPO11 function is activated. Again, the interaction occurred regardless of the presence of nucleic acids, indicating that it occurs (directly or indirectly) between the proteins. Physiologically, SPO11 cleaves DNA at the hotspots marked by H3K4me3; successively, DNA is resected, and the free 3'end becomes the binding site of single-strand binding proteins that promote strand invasion, such as DMC1 [32]. Therefore, if FUS plays a function at hotspots, we expected to find it at sites enriched for both H3K4me3 and DMC1. In accordance our experiments showed that FUS is enriched at autosomal sites 10qC2, and 12qA1.1 selected from a combination of the H3K4me3 and DMC1 ChIP-Seq data set [51], as well as at the 17b and 14a sites, which derive from a combination of PRDM9, H3K4me3, H3K4me36 and DMC1 ChIP-seq experiments [75]. Furthermore, we show that H3K4me36 and PRDM9 are enriched at the autosomal sites 10qC2 and 12qA1.1, although with different magnitudes. We concluded that FUS is localized to DNA at genuine hotspot sites, along with PRDM9. Next, we asked whether FUS could physically interact with SPO11. By a pull-down assay, we found that FUS interacts with SPO11 in vitro, in the absence of testis-specific factors. Moreover, by immunoprecipitating SPO11β from testis extracts, we observed FUS in the immunocomplex in cells in the early meiotic stage in vivo, as there were also PRDM9 and REC114. Notably, although FUS and PRDM9 were found in the immunocomplex with SPO11, their interaction also occurred in the *Spo11*^*−/−*^ testes, indicating that the protein complex forms independently of SPO11 and the formation of DSBs, perhaps in advance of DSBs.

Recent data shows that TOPOVIBL can form a stable complex with REC114, and that this interaction is essential for the formation of DSBs across the genome in the females, while in males it promotes the proper timing of DSB formation, and the placement of DSBs in the subtelomeric regions and in the PAR [69]. As SPO11β and TOPOVIBL are direct binding partners [26], according to the loop/axis-tethering model, we speculate that in females the interaction between TOPOVIBL and REC114 plays a key role in promoting the association of SPO11 with the DSB sites on the axis. In males, in which the lack of interaction between TOPOVIBL and REC114 has a less dramatic effect, FUS by interacting (directly or indirectly) with both PRDM9 and SPO11 may implement the association of SPO11 with the axis, through its (direct or indirect) protein–protein interaction with REC114 (Fig. 4E), while simultaneously adapting PRDM9-tagged sites to the axis. It should be noted that the latter function has previously been assigned to EWS, due to its ability to bind PRDM9, and the proteins associated with the axis REC8 and SYCP3 [53]. Since PRDM9 forms soluble and DNA bound multimeric complexes [79–81], and EWS and FUS are both expressed in early meiotic stages and share the Ranbp2-type zinc finger functional domain, here we propose that FUS and EWS may bind concomitantly to PRDM9 multimers (perhaps separately to the individual monomers of the PRDM9 complex), cooperating to tie PRDM9 to the axis, activating SPO11 function.

In this regard, we found that SPO11 also interacts with EWS in vivo, which supports this hypothesis. Given the substantial severe effect of *Fus* deletion [18] over that of *Ewsr1* [54] on homologous synapsis, we speculate that FUS and EWS may have partially distinct functions, with FUS playing a predominant role in promoting the association of SPO11 with PRDM9 in hotspots, and EWS acting more as a fine-tuning regulator. If this is correct, FUS would replace the function of EWS when the latter is not expressed. This would explain the mild effect of *Ewsr1* deletion in the positioning of hotspots. This could be verified by analyzing the genome-wide distribution of the hotspots in *Fus*^*−/−*^ mice. Unfortunately, the current available *Fus* knockout model dies at perinatal age [82], therefore, at present, direct testing of the hotspot positioning shift in the absence of *Fus* or *Fus* and *Ewsr1* is not possible. In addition to FUS and EWS, PRDM9 has been previously shown to interact directly (in vitro and in vivo) with several other protein factors. Among them, a role at hotspots has so far been assigned to HELLS and speculated for EHMT2 [44, 45, 54]. Such protein factors may interact with PRDM9 with different timings with respect to FUS and EWS, playing distinct functions (supplementary discussion). More studies will be needed to clarify this aspect.

FUS is a complex protein with many functions, including DSB repair mediated by liquid–liquid phase separation [83, 84]. In this regard, the number of DSBs, identified by the RAD51 marker, appears to be very high in *Fus *^*−/−*^ spermatocytes [18], indicating a possible defect in the processing of DSBs. However, a direct involvement of FUS in DSB repair would predict that it is persistent at the site of damage as a punctate staining; a pattern that was not observed in our study. This mitigates the possibility that FUS may play a widespread role in DSB processing steps. In this regard, it is worth underlying that we observed that localization of FUS on the chromosome axis was only partial, suggesting that it is transient. This predicted dynamism of the interaction with the axis reminds of the dynamic location of SPO11, which is expected to be recruited to the axes and released immediately after DSB formation, to prevent its persistent activity on DNA [85].

Interestingly, our data also show that FUS interacts with the SPO11α splice isoform both in vivo and in vitro and localizes at the PAR hotspot, the site of genetic exchange between the XY chromosomes [72]. Therefore, FUS may also play a role in promoting efficient recombination of male-sex chromosomes. This probably occurs independently of its interaction with PRDM9, as the latter is dispensable for the deposition of H3K4me3 at the PAR hotspot [51]. As SPO11α is thought to play a specific function in XY recombination [72], further studies aimed at uncovering other molecular interactors of SPO11α will be instrumental to identify new players involved in the initiation of XY recombination, likely clarifying the function of FUS in this process.

## Materials and methods

### Mice

This study was carried out in accordance with the principles of the Declaration of Helsinki. The approval was granted by the Institutional Animal Care and Use Committee of the University of Rome Tor Vergata and by the “Istituto Superiore di Sanità” of Italy and was carried out according to the guidelines of the committee. *Prdm9*^*−/−*^; *Prdm9*^*Tg/Tg*^* and Prdm9*^*−/−*^; Tg*Prdm9*^*YF/YF*^ mice testes were maintained in C57BL6/J background [47]. *Spo11β*-only and *Spo11α*-only mice were developed in our laboratory by knocking-in either *Spo11β* or *Spo11α* cDNA under the *Spo11* promoter (manuscript in preparation) and maintained in either C57BL6/J or mix (C57BL6/J and 129/Sv) backgrounds. Wild-type mice used in immunoprecipitation and pull-down experiments were C57BL6/J or CD1. Enriched fractions of germ cells and testes somatic cells were obtained from CD1 mice. Wild-type mice used in the ChIP experiments were C57BL6/J. The age and number of testes used in each experiment are specified in the figure legends.

### Preparation of spermatocytes spreads

Spreads were prepared as previously described [42, 61, 86]. Briefly, testes were taken off from juvenile euthanized mice and the tunica albuginea was removed. Next, the seminiferous tubules were placed in DMEM high glucose (Euroclone, ECM0101L) and disrupted and mixed using a razor blade. The supernatant was centrifuged at 7200 rpm for 1 min and the pellet was resuspended in 0.5 M sucrose (VWR, 27480.294). Cell suspension was fixed on slides (Thermo Sientific, Menzel-Glaser Superfrost Plus, J1800AMNZ) using 1% paraformaldehyde (PFA) (ChemCruz, SC-281692)/0.015% Triton X-100 (Sigma-Aldrich, 9002-93-1)/dH2O pH 9.2) and incubated for 2 h in a humidified chamber at room temperature (RT). When the slides were completely fixed, they were washed twice with Washing Buffer 2 [WB2, 0.4% Photo-Flo (Kodak Professional 200 Solution, 1464510)/dH2O] and left air-drying at RT. Slides were either stained soon after or stored at − 80 °C for up to 6 months.

### Immunofluorescence on the meiotic chromosome spreads

Immunofluorescence on surface chromosome spreads was performed as previously described [42, 86]. Briefly, air-dried slides were washed with washing buffer 1 (WB1: 0.4% Photo-Flo, 0.01% Triton X-100/dH2O) and incubated overnight (ON) at room temperature (RT) with primary antibodies in antibody dilution buffer [ADB: 10% goat serum (Sigma-Aldrich, G9023), 3% bovine serum albumin (BSA) (Sigma-Aldrich, A7906), 0.05% Triton X-100 in buffered phosphate (PBS) (Euroclone, ECB4004L)]. The information on the primary antibodies is shown in Table S1. After 10 min of washing with WB1 and WB2, slides were incubated with secondary antibodies for 1 h at 37 °C in the dark using the NeoBrite System (NeoBiotech). Secondary antibodies are listed in Table S1. Following 10 min washes with WB1 and WB2, slides were rinsed for 5 min in PBS and incubated in Hoechst (Thermo Fisher Scientific, 33258)/1X PBS solution for 20 min in a humidified chamber at RT. At the end of the incubation, the slides were air-dried at RT in the dark and mounted using ProLong Gold Antifade mounting media without DAPI (Invitrogen, P36934). Images were captured using a Leica CTR6000 digital inverted microscope connected to a CCD camera. Confocal images were captured using the STEDYCON confocal microscope (Abberior Instruments).

### Fluorescence image colocalization analysis

The colocalization of paired fluorescence signals was estimated by ImageJ using Pearson’s correlation coefficient (PCC). As a control for random colocalization, we performed a scrambling of the FUS channel by rotating the image of 180 degrees.

### Histology and immunostaining of testes sections

Testes were collected and immediately frozen in O.C.T. (Sakura, Tissue-Tek, 4583). Following sectioning, tissues were fixed on glass slides with PFA 4%/PBS for 10 min at RT, followed by 10 min of washing in 1X PBS. Immunostaining was preceded by antigen retrieval using Tris–EDTA citrate buffer, pH 7.8 (UCS Diagnostic, TECH199) for 30 min in steam, followed by cooling to RT. The slides were then rinsed in dH2O to continue with the immunofluorescence assay as previously reported. The images were captured using a STEDYCON confocal microscope (Abberior Instruments).

### Isolation of the enriched somatic and germinal cell population

The isolation of the enriched somatic and germinal cell population was carried out according to Rossi et al. [60]. Briefly, the testes of five 10 dpp wild-type mice were washed in 1X PBS. After removing the supernatant, the testes were resuspended in collagenase (0.5 mg/ml) (Sigma-Aldrich, C7657) for 30 min at 32 °C in agitation in a water bath until the seminiferous tubules were completely dispersed. After allowing the tubules to settle for 5 min, they were washed twice with 1X PBS and digested with 1X Trypsin/EDTA (Aurogene, AU-X0930) for 5 min in agitation at 32 °C. Next, the digestion was blocked with culture media (DMEM high glucose 10% fetal bovine serum [FBS, Sigma-Aldrich, F7524], 1% penicillin/streptomycin [P/S, Euroclone, ECB3001D], 1% L-glutamine [Euroclone, ECB3000D], 1 mM sodium pyruvate [Euroclone, ECM0542D] and 2 mM sodium lactate [Sigma-Aldrich, L-7900]). Cells were then spun down and resuspended in a small volume of culture medium supplemented with 1X DNAse (Sigma-Aldrich, DN25). The suspension was cultured in culture medium in a 5% CO2 atmosphere at 32 °C for 3 h. At the end of incubation, floating cells, which is the fraction of enriched germ cells, were collected, washed with 1X PBS, and the pellet stored at − 80 °C until use. Cells in adhesion, which are the enriched fraction of testes somatic cells, were washed twice to remove the culture medium and treated with a hypotonic solution (20 mM Tris HCl pH 7.5) for 2 min to remove residual germinal cells and cultured for 24 h in high glucose DMEM without FBS. The following day, cells were collected, washed with 1X PBS, and the pellets were stored at − 80 °C until use.

### Centrifugal elutriation

The isolation of enriched populations of male mouse germ cells was performed using centrifugal elutriation according to Barchi et al. [61].

### Immunoprecipitation and Western Blot

Immunoprecipitation was performed according to [54] with minor changes. Briefly, testes from adult or juvenile wild type mice were decapsulated and lysed using the Pierce IP Lysis Buffer (Thermo Fisher Scientific, 87787) complemented with proteases inhibitors 2X (Roche, cOmplete Tablets EDTA-free, 04693132001), phosphatases Inhibitors 1X (Sigma-Aldrich, Phosphatase Inhibitor Cocktail 3, P0044) and benzonase (ChemCruz, sc-202391A) according to manufacturer instructions. Supernatants were incubated with Dynabeads Protein-A (Thermo Fisher Scientific, 1002D) loaded with either the mouse monoclonal anti-SPO11-180 antibody (table S1), which recognizes specifically both SPO11β and SPO11α isoforms [85, 86], or anti-FUS antibody, which specifically recognizes FUS protein [87], in rotation at 4 °C. Mouse anti-IgG2A (table S1) served as a control. At the end of incubation, the dynabeads were washed three times with Lysis buffer and eluted with standard Laemmli buffer. The samples were fractionated on 8–12% SDS-PAGE and transferred to a PVDF membrane (GE Healthcare, Amersham Hybond P Western blotting membranes, GE10600023) using a semi-dry transfer system (Hoefer, TE22). For Western Blot (WB) analysis, membranes were probed with primary antibodies diluted in BSA 5%/TBS 0.1% Tween 20 (TBS-T). Secondary antibodies were diluted in 5% nonfat dry milk (AppliChem, A0830)/TBS-T. Primary and secondary antibodies are listed in Table S1. WB signals were detected using ECL reagent (BIO-RAD, Clarity Western ECL Substrate, #170-5061).

### Pulldown assay

Spo11β and Spo11α were cloned into a pGEX-4T1 plasmid (Addgene). PRDM9 was cloned in a pMAL-C5x plasmid (New England Biolabs), gifted by Petko Petkov. The plasmids pGEX-4T1 Spo11α and pGEX-4T1 Spo11β and pMAL-C5X PRDM9 were transformed by heat shock into E. coli BL21. Transformed cells were grown in LB medium, supplemented with 50 μg/mL Ampicillin (Sigma-Aldrich, A0166), till an O.D. (600 nm) of 0.6–0.7 and induced with 0.5 mM isopropyl-thiogalactoside (IPTG) for protein expression, at 37 °C for 3 h. For GST-recombinant protein purification, each pellet was resuspended in GST binding buffer (PBS/1% Triton X-100 + protease inhibitors + DTT 1 mM), sonicated, and centrifugated at 12,000 rpm for 30 min at 4 °C. Recombinant proteins were purified by incubating lysates with glutathione-agarose beads (Sigma-Aldrich, #SLBX5204), at 4 °C for 2 h under constant rotation. The beads were washed twice in GST binding buffer and used in the pull-down experiments. For the GST pull-down assay, testes were lysed in Pierce Lysis Buffer (Thermo Fisher Scientific, 87787) supplemented with protease inhibitors, phosphatases inhibitors, and benzonase, while HEK293 cells were lysed in HEK-lysis buffer (400 mM NaCl, 5 mM EDTA, 50 mM Hepes–NaOH ph 7.4, 1% TritonX-100) with protease inhibitors. The lysates were incubated with GST-Spo11α/β-beads for 3 h at 4 °C. After washing with PBS 1X, proteins were eluted in Laemmli buffer 2X by heating to 95 °C for 5 min. Proteins were analyzed by WB. For PRDM9-MBP purification, each pellet was resuspended in MBP column binding buffer (20 mM Tris–HCl PH 7.4, 1 mM EDTA, 100 mM NaCl + proteases inhibitors + DTT 1 mM), sonicated, and centrifugated at 12,000 rpm for 30 min at 4 °C. Recombinant proteins were purified by incubating lysates with Amylose resin (NEB, #E802S) at 4 °C for 2 h under constant rotation. The beads were washed twice in MBP column binding buffer and used in the pull-down experiments. For MBP-pull down assay, testes and HEK293 cells were lysed in Pierce Lysis Buffer and HEK-lysis buffer, respectively, and incubated with PRDM9-MBP-beads for 3 h at 4 °C. After washing with PBS 1X, proteins were eluted in Laemmli buffer 2X by heating to 95 °C for 5 min. Proteins were analyzed by WB.

### Chromatin immunoprecipitation (ChIP)

#### Crosslinking from mouse testes chromatin

Testes were taken off from juvenile euthanized male mice and the tunica albuginea was removed. The seminiferous tubules were placed in 1X PBS on ice and disrupted and mixed using a razor blade. Spermatocyte suspension was resuspended in 1X PBS and left on ice to settle for 10 min. The supernatant was centrifuged at 2000 rpm for 5 min. The pellet was resuspended in 1X PBS and then 1% formaldehyde was added to the resuspension of the cells and incubated for 10 min at RT. The crosslinking reaction was stopped using 0.125 M Glycine (SERVA, 23391.02) for 5 min at RT. Cell resuspension was washed twice with cold 1X PBS and centrifuged at 2000 rpm for 5 min. The supernatant was removed and the pellet was sonicated or stored at − 80 °C after freezing in liquid nitrogen.

#### Immunoprecipitation

The pellet was resuspended with nuclear extraction buffer (5 mM Pipes pH 8; 85 mM KCl; 0.5% NP-40, mM dithiothreitol, 10 mM β-glycerophosphate, 0.5 mM Na3VO4, and protease inhibitor cocktail–Sigma Aldrich), incubated on ice for 10 min and centrifuged at 1000×*g* for 10 min at 4 °C. The pellet was resuspended in 250 μl Lysis buffer (1% SDS, 10 mM EDTA pH 8, 50 mM Tris HCl pH 8, mM dithiothreitol, 10 mM β-glycerophosphate, 0.5 mM Na3VO4 and protease inhibitor cocktail) and incubated on ice for 10 min. The samples were sonicated with Bioruptor (Dyagenode) 2 × 5 min (30-s sonication and 30-s pause) and centrifuged at 10,000×*g* for 10 min at 4 °C. The DNA was quantified using Nanodrop and checked with electrophoresis run using a 1% agarose gel. The samples were pre-cleared for 2 h with protein A/agarose/salmon sperm DNA (Millipore) and then immunoprecipitated ON using 1 µg of anti-H3K4me3 and anti-FUS antibody (table S1) in constant rotation. The beads were washed four times with the low salt buffer A (0.1% SDS, 1% Triton X-100, 2 mM EDTA pH 8, 20 mM Tris HCl pH 8, 150 mM NaCl, with protease inhibitors) and three times with the high salt buffer B (01% SDS, 1% Triton X-100, 2 mM EDTA pH 8, 20 mM Tris HCl pH 8, 500 mM NaCl, with protease inhibitors) on ice. The last wash was performed using Tris–EDTA buffer (10 mM Tris–HCl and 1 mM EDTA pH 8) and the samples were incubated for 5 min at RT. The supernatant was then discarded and the beads were eluted twice using elution buffer (1% SDS and 0.1 M NaHCO_3_) and incubated for 15 min at RT. Finally, in each sample, we added NaCl to 0.2 M and incubated them at 65 °C ON. The next day, the Proteinase K solution (0.5 M EDTA, 1 M Tris HCl pH 6.8, PK 0.025 µg/ml) had been added to each sample and incubated and stirred for 1 h at 45 °C.

#### DNA extraction

Each sample was treated with an equal volume of phenol–chloroform-isoamyl alcohol (Thermo Fisher Scientific) and centrifuged at 13,000 rpm for 10 min at 4 °C. Sodium acetate (3 M, pH 5.2), glycogen (Thermo Fisher Scientific) (100 µg/µl), and EtOH 100% were added to the supernatant, and it has been left to precipitate ON at − 80 °C. Then it was centrifuged at 13,000 rpm for 40 min at 4 °C. The pellet was washed with EtOH 75% and, after rapid centrifugation, dried at 50 °C for 3 min and resuspended with dH2O at 50 °C for 5 min. The DNA obtained was analyzed by qPCR. The primers are listed in Table S2.

### Statistical analysis

Statistical analysis was performed with GraphPad Prism 9 for Macintosh (GraphPad Software, San Diego, CA). Data were expressed as mean ± SD or mean ± SEM, as detailed in the figure captions.

### Artwork

The artwork was done with Adobe Photoshop and Illustrator 2022.

## Fundings

This work was supported by the Telethon Foundation, Grant no. GGP12189 (MB); University of Rome Tor Vergata, Grant “Consolidate the Fundations”, Grant no. 141 (MB); Regione Lazio, Italy, Grant “Progetti Gruppi di Ricerca 2020” POR FESR Lazio 2014-2020, Grant no. A0375-2020-36618 (MB and MPP). The authors declare that no funds, grants, or other support were received during the preparation of this manuscript.

### Supplementary Information

Below is the link to the electronic supplementary material.

Supplementary file1 (DOCX 38 KB)

Supplementary file2 (PDF 1334 KB)

Supplementary file3 (PDF 16631 KB)

Supplementary file4 (PDF 85 KB)

Supplementary file5 (DOCX 23 KB)

## Data Availability

The authors confirm that the data supporting the findings of this study are available in the article and its supplementary materials.

## References

[1] Svetoni F, Frisone P, Paronetto MP (2016). Role of FET proteins in neurodegenerative disorders. RNA Biol.

[2] Andersson MK, Stahlberg A, Arvidsson Y, Olofsson A, Semb H, Stenman G (2008). The multifunctional FUS, EWS and TAF15 proto-oncoproteins show cell type-specific expression patterns and involvement in cell spreading and stress response. BMC Cell Biol.

[3] Tan AY, Manley JL (2009). The TET family of proteins: functions and roles in disease. J Mol Cell Biol.

[4] Paronetto MP (2013). Ewing sarcoma protein: a key player in human cancer. Int J Cell Biol..

[5] Bertolotti A, Lutz Y, Heard DJ, Chambon P, Tora L (1996). hTAF(II)68, a novel RNA/ssDNA-binding protein with homology to the pro-oncoproteins TLS/FUS and EWS is associated with both TFIID and RNA polymerase II. EMBO J.

[6] Yang L, Embree LJ, Tsai S, Hickstein DD (1998). Oncoprotein TLS interacts with serine-arginine proteins involved in RNA splicing. J Biol Chem.

[7] Lerga A, Hallier M, Delva L, Orvain C, Gallais I, Marie J (2001). Identification of an RNA binding specificity for the potential splicing factor TLS. J Biol Chem.

[8] Dutertre M, Sanchez G, De Cian MC, Barbier J, Dardenne E, Gratadou L (2010). Cotranscriptional exon skipping in the genotoxic stress response. Nat Struct Mol Biol.

[9] Paronetto MP, Bernardis I, Volpe E, Bechara E, Sebestyen E, Eyras E (2014). Regulation of FAS exon definition and apoptosis by the Ewing sarcoma protein. Cell Rep.

[10] Baechtold H, Kuroda M, Sok J, Ron D, Lopez BS, Akhmedov AT (1999). Human 75-kDa DNA-pairing protein is identical to the pro-oncoprotein TLS/FUS and is able to promote D-loop formation. J Biol Chem.

[11] Paronetto MP, Minana B, Valcarcel J (2011). The Ewing sarcoma protein regulates DNA damage-induced alternative splicing. Mol Cell.

[12] Lee SG, Kim N, Kim SM, Park IB, Kim H, Kim S (2020). Ewing sarcoma protein promotes dissociation of poly(ADP-ribose) polymerase 1 from chromatin. EMBO Rep.

[13] Aleksandrov R, Dotchev A, Poser I, Krastev D, Georgiev G, Panova G (2018). Protein dynamics in complex DNA lesions. Mol Cell.

[14] Mastrocola AS, Kim SH, Trinh AT, Rodenkirch LA, Tibbetts RS (2013). The RNA-binding protein fused in sarcoma (FUS) functions downstream of poly(ADP-ribose) polymerase (PARP) in response to DNA damage. J Biol Chem.

[15] Sebesta M, Burkovics P, Juhasz S, Zhang S, Szabo JE, Lee MY (2013). Role of PCNA and TLS polymerases in D-loop extension during homologous recombination in humans. DNA Repair (Amst).

[16] Tian H, Petkov PM (2021). Mouse EWSR1 is crucial for spermatid post-meiotic transcription and spermiogenesis. Development.

[17] Li H, Watford W, Li C, Parmelee A, Bryant MA, Deng C (2007). Ewing sarcoma gene EWS is essential for meiosis and B lymphocyte development. J Clin Invest.

[18] Kuroda M, Sok J, Webb L, Baechtold H, Urano F, Yin Y (2000). Male sterility and enhanced radiation sensitivity in TLS(-/-) mice. EMBO J.

[19] Oakberg EF (1956). Duration of spermatogenesis in the mouse and timing of stages of the cycle of the seminiferous epithelium. Am J Anat.

[20] Barchi M, Mahadevaiah S, Di Giacomo M, Baudat F, de Rooij DG, Burgoyne PS (2005). Surveillance of different recombination defects in mouse spermatocytes yields distinct responses despite elimination at an identical developmental stage. Mol Cell Biol.

[21] Russell LE, Hikim APS, Clegg ED (1990). Histological and histopatological evaluation of the testis.

[22] Boateng KA, Bellani MA, Gregoretti IV, Pratto F, Camerini-Otero RD (2013). Homologous pairing preceding SPO11-mediated double-strand breaks in mice. Dev Cell.

[23] Sole M, Blanco J, Gil D, Valero O, Cardenas B, Fonseka G (2022). Time to match; when do homologous chromosomes become closer?. Chromosoma.

[24] Keeney S (2001). Mechanism and control of meiotic recombination initiation. Curr Top Dev Biol.

[25] Keeney S (2008). Spo11 and the formation of DNA double-strand breaks in meiosis. Genome Dyn Stab.

[26] Robert T, Nore A, Brun C, Maffre C, Crimi B, Bourbon HM (2016). The TopoVIB-Like protein family is required for meiotic DNA double-strand break formation. Science.

[27] Mahadevaiah SK, Turner JM, Baudat F, Rogakou EP, de Boer P, Blanco-Rodriguez J (2001). Recombinational DNA double-strand breaks in mice precede synapsis. Nat Genet.

[28] Baudat F, Manova K, Yuen JP, Jasin M, Keeney S (2000). Chromosome synapsis defects and sexually dimorphic meiotic progression in mice lacking Spo11. Mol Cell.

[29] Romanienko PJ, Camerini-Otero RD (2000). The mouse Spo11 gene is required for meiotic chromosome synapsis. Mol Cell.

[30] Page SL, Hawley RS (2004). The genetics and molecular biology of the synaptonemal complex. Annu Rev Cell Dev Biol.

[31] Guillon H, Baudat F, Grey C, Liskay RM, de Massy B (2005). Crossover and noncrossover pathways in mouse meiosis. Mol Cell.

[32] La Volpe A, Barchi M (2012). Meiotic double strand breaks repair in sexually reproducing eukaryotes: we are not all equal. Exp Cell Res.

[33] Lorenz A, Estreicher A, Kohli J, Loidl J (2006). Meiotic recombination proteins localize to linear elements in Schizosaccharomyces pombe. Chromosoma.

[34] Miyoshi T, Ito M, Kugou K, Yamada S, Furuichi M, Oda A (2012). A central coupler for recombination initiation linking chromosome architecture to S phase checkpoint. Mol Cell.

[35] Kumar R, Bourbon HM, de Massy B (2010). Functional conservation of Mei4 for meiotic DNA double-strand break formation from yeasts to mice. Genes Dev.

[36] Stanzione M, Baumann M, Papanikos F, Dereli I, Lange J, Ramlal A (2016). Meiotic DNA break formation requires the unsynapsed chromosome axis-binding protein IHO1 (CCDC36) in mice. Nat Cell Biol.

[37] Kumar R, Oliver C, Brun C, Juarez-Martinez AB, Tarabay Y, Kadlec J (2018). Mouse REC114 is essential for meiotic DNA double-strand break formation and forms a complex with MEI4. Life Sci Alliance..

[38] Reinholdt LG, Schimenti JC (2005). Mei1 is epistatic to Dmc1 during mouse meiosis. Chromosoma.

[39] Acquaviva L, Boekhout M, Karasu ME, Brick K, Pratto F, Li T (2020). Ensuring meiotic DNA break formation in the mouse pseudoautosomal region. Nature.

[40] Libby BJ, De La Fuente R, O'Brien MJ, Wigglesworth K, Cobb J, Inselman A (2002). The mouse meiotic mutation mei1 disrupts chromosome synapsis with sexually dimorphic consequences for meiotic progression. Dev Biol.

[41] Kauppi L, Barchi M, Lange J, Baudat F, Jasin M, Keeney S (2013). Numerical constraints and feedback control of double-strand breaks in mouse meiosis. Genes Dev.

[42] Faieta M, Di Cecca S, de Rooij DG, Luchetti A, Murdocca M, Di Giacomo M (2016). A surge of late-occurring meiotic double-strand breaks rescues synapsis abnormalities in spermatocytes of mice with hypomorphic expression of SPO11. Chromosoma.

[43] Baudat F, Buard J, Grey C, Fledel-Alon A, Ober C, Przeworski M (2010). PRDM9 is a major determinant of meiotic recombination hotspots in humans and mice. Science.

[44] Imai Y, Biot M, Clement JA, Teragaki M, Urbach S, Robert T (2020). PRDM9 activity depends on HELLS and promotes local 5-hydroxymethylcytosine enrichment. Elife.

[45] Spruce C, Dlamini S, Ananda G, Bronkema N, Tian H, Paigen K (2020). HELLS and PRDM9 form a pioneer complex to open chromatin at meiotic recombination hot spots. Genes Dev.

[46] Powers NR, Parvanov ED, Baker CL, Walker M, Petkov PM, Paigen K (2016). The meiotic recombination activator PRDM9 trimethylates both H3K36 and H3K4 at recombination hotspots in vivo. PLoS Genet..

[47] Diagouraga B, Clement JAJ, Duret L, Kadlec J, de Massy B, Baudat F (2018). PRDM9 methyltransferase activity is essential for meiotic DNA double-strand break formation at its binding sites. Mol Cell.

[48] Grey C, Barthes P, Chauveau-Le Friec G, Langa F, Baudat F, de Massy B (2011). Mouse PRDM9 DNA-binding specificity determines sites of histone H3 lysine 4 trimethylation for initiation of meiotic recombination. PLoS Biol..

[49] Parvanov ED, Petkov PM, Paigen K (2010). Prdm9 controls activation of mammalian recombination hotspots. Science.

[50] Myers S, Bowden R, Tumian A, Bontrop RE, Freeman C, MacFie TS (2010). Drive against hotspot motifs in primates implicates the PRDM9 gene in meiotic recombination. Science.

[51] Brick K, Smagulova F, Khil P, Camerini-Otero RD, Petukhova GV (2012). Genetic recombination is directed away from functional genomic elements in mice. Nature.

[52] Sun F, Fujiwara Y, Reinholdt LG, Hu J, Saxl RL, Baker CL (2015). Nuclear localization of PRDM9 and its role in meiotic chromatin modifications and homologous synapsis. Chromosoma.

[53] Tian H, Billings T, Petkov PM (2021). EWSR1 affects PRDM9-dependent histone 3 methylation and provides a link between recombination hotspots and the chromosome axis protein REC8. Mol Biol Cell.

[54] Parvanov ED, Tian H, Billings T, Saxl RL, Spruce C, Aithal R (2017). PRDM9 interactions with other proteins provide a link between recombination hotspots and the chromosomal axis in meiosis. Mol Biol Cell.

[55] Panizza S, Mendoza MA, Berlinger M, Huang L, Nicolas A, Shirahige K (2011). Spo11-accessory proteins link double-strand break sites to the chromosome axis in early meiotic recombination. Cell.

[56] ClaeysBouuaert C, Pu S, Wang J, Oger C, Daccache D, Xie W (2021). DNA-driven condensation assembles the meiotic DNA break machinery. Nature.

[57] Blat Y, Protacio RU, Hunter N, Kleckner N (2002). Physical and functional interactions among basic chromosome organizational features govern early steps of meiotic chiasma formation. Cell.

[58] de Massy B (2013). Initiation of meiotic recombination: how and where? Conservation and specificities among eukaryotes. Annu Rev Genet.

[59] Tarsounas M, Morita T, Pearlman RE, Moens PB (1999). RAD51 and DMC1 form mixed complexes associated with mouse meiotic chromosome cores and synaptonemal complexes. J Cell Biol.

[60] Rossi P, Dolci S, Albanesi C, Grimaldi P, Ricca R, Geremia R (1993). Follicle-stimulating hormone induction of steel factor (SLF) mRNA in mouse Sertoli cells and stimulation of DNA synthesis in spermatogonia by soluble SLF. Dev Biol.

[61] Barchi M, Geremia R, Magliozzi R, Bianchi E (2009). Isolation and analyses of enriched populations of male mouse germ cells by sedimentation velocity: the centrifugal elutriation. Methods Mol Biol.

[62] Ahmed EA, de Rooij DG (2009). Staging of mouse seminiferous tubule cross-sections. Methods Mol Biol.

[63] Dobson MJ, Pearlman RE, Karaiskakis A, Spyropoulos B, Moens PB (1994). Synaptonemal complex proteins: occurrence, epitope mapping and chromosome disjunction. J Cell Sci.

[64] Ashley T, Gaeth AP, Creemers LB, Hack AM, de Rooij DG (2004). Correlation of meiotic events in testis sections and microspreads of mouse spermatocytes relative to the mid-pachytene checkpoint. Chromosoma.

[65] Turner JM (2007). Meiotic sex chromosome inactivation. Development.

[66] Paigen K, Petkov PM (2018). PRDM9 and its role in genetic recombination. Trends Genet.

[67] Boekhout M, Karasu ME, Wang J, Acquaviva L, Pratto F, Brick K (2019). REC114 partner ANKRD31 controls number, timing, and location of meiotic DNA breaks. Mol Cell.

[68] Papanikos F, Clement JAJ, Testa E, Ravindranathan R, Grey C, Dereli I (2019). Mouse ANKRD31 regulates spatiotemporal patterning of meiotic recombination initiation and ensures recombination between X and Y sex chromosomes. Mol Cell.

[69] Nore A, Juarez-Martinez AB, Clement J, Brun C, Diagouraga B, Laroussi H (2022). TOPOVIBL-REC114 interaction regulates meiotic DNA double-strand breaks. Nat Commun.

[70] Keeney S, Baudat F, Angeles M, Zhou ZH, Copeland NG, Jenkins NA (1999). A mouse homolog of the Saccharomyces cerevisiae meiotic recombination DNA transesterase Spo11p. Genomics.

[71] Romanienko PJ, Camerini-Otero RD (1999). Cloning, characterization, and localization of mouse and human SPO11. Genomics.

[72] Kauppi L, Barchi M, Baudat F, Romanienko PJ, Keeney S, Jasin M (2011). Distinct properties of the XY pseudoautosomal region crucial for male meiosis. Science.

[73] Billings T, Parvanov ED, Baker CL, Walker M, Paigen K, Petkov PM (2013). DNA binding specificities of the long zinc-finger recombination protein PRDM9. Genome Biol.

[74] Crichton JH, Playfoot CJ, MacLennan M, Read D, Cooke HJ, Adams IR (2017). Tex19.1 promotes Spo11-dependent meiotic recombination in mouse spermatocytes. PLoS Genet..

[75] Grey C, Clement JA, Buard J, Leblanc B, Gut I, Gut M (2017). In vivo binding of PRDM9 reveals interactions with noncanonical genomic sites. Genome Res.

[76] Keeney S, Lange J, Mohibullah N (2014). Self-organization of meiotic recombination initiation: general principles and molecular pathways. Annu Rev Genet.

[77] Hunter N (2015). Meiotic recombination: the essence of heredity. Cold Spring Harb Perspect Biol.

[78] Steggerda SM, Paschal BM (2002). Regulation of nuclear import and export by the GTPase Ran. Int Rev Cytol.

[79] Baker CL, Petkova P, Walker M, Flachs P, Mihola O, Trachtulec Z (2015). Multimer formation explains allelic suppression of PRDM9 recombination hotspots. PLoS Genet..

[80] Altemose N, Noor N, Bitoun E, Tumian A, Imbeault M, Chapman JR (2017). A map of human PRDM9 binding provides evidence for novel behaviors of PRDM9 and other zinc-finger proteins in meiosis. Elife.

[81] Schwarz T, Striedner Y, Horner A, Haase K, Kemptner J, Zeppezauer N (2019). PRDM9 forms a trimer by interactions within the zinc finger array. Life Sci Alliance..

[82] Hicks GG, Singh N, Nashabi A, Mai S, Bozek G, Klewes L (2000). Fus deficiency in mice results in defective B-lymphocyte development and activation, high levels of chromosomal instability and perinatal death. Nat Genet.

[83] Sukhanova MV, Singatulina AS, Pastre D, Lavrik OI (2020). Fused in sarcoma (FUS) in DNA repair: tango with poly(ADP-ribose) polymerase 1 and compartmentalisation of damaged DNA. Int J Mol Sci.

[84] Spegg V, Altmeyer M (2021). Biomolecular condensates at sites of DNA damage: more than just a phase. DNA Repair (Amst)..

[85] Neale MJ, Pan J, Keeney S (2005). Endonucleolytic processing of covalent protein-linked DNA double-strand breaks. Nature.

[86] Testa E, Nardozi D, Antinozzi C, Faieta M, Di Cecca S, Caggiano C (2018). H2AFX and MDC1 promote maintenance of genomic integrity in male germ cells. J Cell Sci.

[87] Ruggiero E, Frasson I, Tosoni E, Scalabrin M, Perrone R, Marusic M (2022). Fused in liposarcoma protein, a new player in the regulation of HIV-1 transcription, binds to known and newly identified LTR G-quadruplexes. ACS Infect Dis.

