# Supplementary material for: The RNA-binding protein FUS/TLS interacts with SPO11 and PRDM9 and localize at meiotic recombination hotspots
Source: Cell Mol Life Sci. 2023 Mar 26;80(4):107. doi: 10.1007/s00018-023-04744-5 (PMC10040399; doi:10.1007/s00018-023-04744-5)
Supplement: Supplementary file 1 — Supplementary file1 (DOCX 38 KB) [file 18_2023_4744_MOESM1_ESM.docx]

***Figures and Captions, Supplementary Discussion***

**THE RNA-BINDING PROTEIN FUS/TLS INTERACTS WITH SPO11 AND PROVIDES A LINK WITH PRDM9-DEPENDENT RECOMBINATION HOTSPOTS**

Teresa Giannattasio^1^, Erika Testa^1,^ °, Ramona Palombo^2,^ °, Lidia Chellini^2,^ °, Flavia Franceschini^1^, Álvaro Crevenna^3^, Petko M. Petkov^4^, Maria Paola Paronetto^2, 5, *^ and Marco Barchi^1, 7, *^

^1^ University of Rome “Tor Vergata”, section of anatomy, via Montpellier, 1, 00133 Rome, Italy; ^2^ Laboratory of Molecular and Cellular Neurobiology, Fondazione Santa Lucia, CERC, 00143 Rome, Italy; ^3^ European Molecular Biology Laboratory, Rome, Italy ^4^ The Jackson Laboratory, Bar Harbor, ME 04609, USA; ^5^ Institut de Génétique Humaine (IGH), University of Montpellier, Centre National de la Recherche Scientifique, Montpellier, France; ^6^ Department of Movement, Human and Health Sciences, University of Rome Foro Italico, Piazza Lauro de Bosis 6, 00135 Rome, Italy.

Correspondence: [mariapaola.paronetto@uniroma4.it](mailto:mariapaola.paronetto@uniroma4.it) (MPP); [marco.barchi@uniroma2.it](mailto:marco.barchi@uniroma2.it) (MB)

°These authors contributed equally to this work

* Corresponding author

7 Lead contact

**Fig. S1** A) Representative images of zygonema cells, stained by SYCP3 and FUS, acquired using inverted fluorescence microscopy. Magnifications show single channels of SYCP3 (green) and FUS (red). Harrows point to the FUS foci and aggregates that co-occur with SYCP3. B-C) Representative image of a cell at preleptonema/leptonema stained by SYCP3 and FUS and acquired by confocal microscopy. Single channels are in grey, while in merge SYCP3 is red and FUS is grey. In the bottom panel the FUS channel was rotated clockwise of 180 degrees (flip 180) and overlapped with SYCP3. D-E) Red dots, Pearson’s colocalization coefficient (PCC) of FUS/SYCP3 calculated in cells at the indicated stage of development. Black dots, PCC calculated in the same set of cells after clockwise rotation of the FUS channel of 180 degrees (***p<0.001). F) Representative image of SYCP3 and FUS pattern at pachynema. Magnification bars represent 10 μm.

**Fig. S2** A) Representative images of SYCP3 (red) and PRDM9 (gray) staining in spermatocytes at the indicated stages of development. Magnification bar represents 10 μm. B) quantification of PRDM9/SYCP3 colocalization and C) FUS/PRDM9 co-localization by Pearson’s correlation coefficient (PCC) calculated in a representative group of cells at the indicated stages of development, before (red dots) and after (black dots) rotation of the PRDM9 channel. Each dot is a cell. Error bars are mean ± standard error of the mean. Statistical significances (***p<0.001) are calculated by the two-tailed t test. D) percentage of prophase I stage cells in the testes of 10 dpp mice testes; preleptotene (pLe), leptotene (Le), early zygotene (eZ), mid-zygotene (mZ), late-zygotene (lZ), and early pachytene (eP) substages were quantified. The number of cells (n) is indicated of each substage. E) IP of SPO11 and WB of PRDM9, EWSR1and co-immunoprecipitating from total testes extracts of juvenile (12dpp) mice and isolated populations of germ cells. Pach/Dip and rSpe are pachytene/diplotene-enriched fractions and round spermatids, respectively, obtained from adult mice. The IP with a nonspecific mouse IgG from a 6-month-old mouse, served as a negative control. IP of SPO11 from a purified fraction of r-Spe served as a control for the specificity of the PRDM9 and REC114 antibodies. F) IP/WB analysis of FUS and WB analysis of FUS and PRDM9, in total testes extract from adult *Spo11^-/-^* mice.

**Fig. S3** Enrichment of PRDM9, H3K4me3, H3K36me3 and DMC1 at the indicated hotspots and coldspots.

**Supplementary Discussion**

Among protein factors that have been found to interact and being recruited to hotspots trough PRDM9, is included HELLS. The latter was found to interact with PRDM9 labeled with Nt and Ct in HeLa S3 cells [1] and coimmunoprecipitate in juvenile testes extracts [2]. HELLS promotes efficient binding of PRDM9 to its sites and, by changing the epigenetic state of the chromatin, makes it accessible to the DSB machinery [1]. It is reasonable that, through the formation of PRDM9 multimers, HELLS is recruited, along with FUS/EWS at PRDM9-dependent hotspots, promoting changing of the epigenetic state of the chromatin and access of SPO11, with consequent interaction of FUS/EWS with SPO11. Alternatively, the interaction of PRDM9 with HELLS and FUS/EWS at hotspots may be temporarily separated, with the formation of the PRDM9-HELLS complex preceding that between PRDM9 and FUS/EWS.

EHMT2 (also known as G92a KMT1C), was found to interact with PRDM9 *in vitro* and *in vivo* [3]. Its function is crucial for exerting H3K9 mono- and di-methyltransferase which mainly localizes at heterochromatin and contributes to transcriptional silencing. EHMT2 is expressed in spermatogonia and in the early stages of leptotene, and loss of *Emht2* in mice negatively affects the pairing of homologous chromosomes during meiotic prophase, recapitulating one of the phenotypic characteristics of *Prdm9^-/-^* mice. However, whether gene loss impacts hotspot determination has not been directly tested. For this protein, a possible role has been proposed, along with CDYL, in the definition of the dimensions of hotspots by limiting the lateral extent of nucleosome trimethylation by PRDM9 (see [3] and references therein). Importantly, EHMT2, along with CDYL, has been proposed to form a separate complex with PRDM9 from the one with EWSR1 [3]; therefore, EHMT2 and CDYL may act with PRDM9, with a different timing from EWS and FUS.

CXXC1 also interact with PRDM9 *in vivo* and *in vitro* [3]. However, opposite to *Prdm9^-/-^* mice [4, 5], *Cxxc1^-/-^* mice spermatocytes did not show massive defects in homolog synapsis [6, 7]. Therefore, it is unlikely that *Cxxc1* plays an important role in the designation of hotspots in mammals, even not in mice with C57 background where PRDM9 plays a key function in meiosis. Accordingly, *Cxxc1* has been attributed a function from pachynema onward, in promoting the sheltering of DSBs, crossover formation, and gene expression [7]. In this regard, PRDM9 has recently been proposed to play a role in local processing of SPO11-mediated DSBs [8]. Therefore, it is possible that the interaction between PRDM9 and CXXC1 is necessary for implementing this later function, rather than for the determination of the hotspots.

References:

1. Imai Y, Biot M, Clement JA, Teragaki M, Urbach S, Robert T, et al. PRDM9 activity depends on HELLS and promotes local 5-hydroxymethylcytosine enrichment. Elife. 2020;9. Epub 2020/10/14. doi: 10.7554/eLife.57117. PubMed PMID: 33047671; PubMed Central PMCID: PMCPMC7599071.

2. Spruce C, Dlamini S, Ananda G, Bronkema N, Tian H, Paigen K, et al. HELLS and PRDM9 form a pioneer complex to open chromatin at meiotic recombination hot spots. Genes Dev. 2020;34(5-6):398-412. Epub 2020/02/01. doi: 10.1101/gad.333542.119. PubMed PMID: 32001511; PubMed Central PMCID: PMCPMC7050486.

3. Parvanov ED, Tian H, Billings T, Saxl RL, Spruce C, Aithal R, et al. PRDM9 interactions with other proteins provide a link between recombination hotspots and the chromosomal axis in meiosis. Mol Biol Cell. 2017;28(3):488-99. Epub 2016/12/10. doi: 10.1091/mbc.E16-09-0686. PubMed PMID: 27932493; PubMed Central PMCID: PMCPMC5341731.

4. Brick K, Smagulova F, Khil P, Camerini-Otero RD, Petukhova GV. Genetic recombination is directed away from functional genomic elements in mice. Nature. 2012;485(7400):642-5. Epub 2012/06/05. doi: 10.1038/nature11089. PubMed PMID: 22660327; PubMed Central PMCID: PMCPMC3367396.

5. Sun F, Fujiwara Y, Reinholdt LG, Hu J, Saxl RL, Baker CL, et al. Nuclear localization of PRDM9 and its role in meiotic chromatin modifications and homologous synapsis. Chromosoma. 2015;124(3):397-415. Epub 2015/04/22. doi: 10.1007/s00412-015-0511-3. PubMed PMID: 25894966; PubMed Central PMCID: PMCPMC4550572.

6. Tian H, Billings T, Petkov PM. CXXC1 is not essential for normal DNA double-strand break formation and meiotic recombination in mouse. PLoS Genet. 2018;14(10):e1007657. Epub 2018/10/27. doi: 10.1371/journal.pgen.1007657. PubMed PMID: 30365547; PubMed Central PMCID: PMCPMC6221362.

7. Jiang Y, Zhang HY, Lin Z, Zhu YZ, Yu C, Sha QQ, et al. CXXC finger protein 1-mediated histone H3 lysine-4 trimethylation is essential for proper meiotic crossover formation in mice. Development. 2020;147(6). Epub 2020/02/26. doi: 10.1242/dev.183764. PubMed PMID: 32094118.

8. Paiano J, Wu W, Yamada S, Sciascia N, Callen E, Paola Cotrim A, et al. ATM and PRDM9 regulate SPO11-bound recombination intermediates during meiosis. Nat Commun. 2020;11(1):857. Epub 2020/02/14. doi: 10.1038/s41467-020-14654-w. PubMed PMID: 32051414; PubMed Central PMCID: PMCPMC7016097.
