# Supplementary figures and images for: The RNA-binding protein FUS/TLS interacts with SPO11 and PRDM9 and localize at meiotic recombination hotspots
Source: Cell Mol Life Sci. 2023 Mar 26;80(4):107. doi: 10.1007/s00018-023-04744-5 (PMC10040399; doi:10.1007/s00018-023-04744-5)

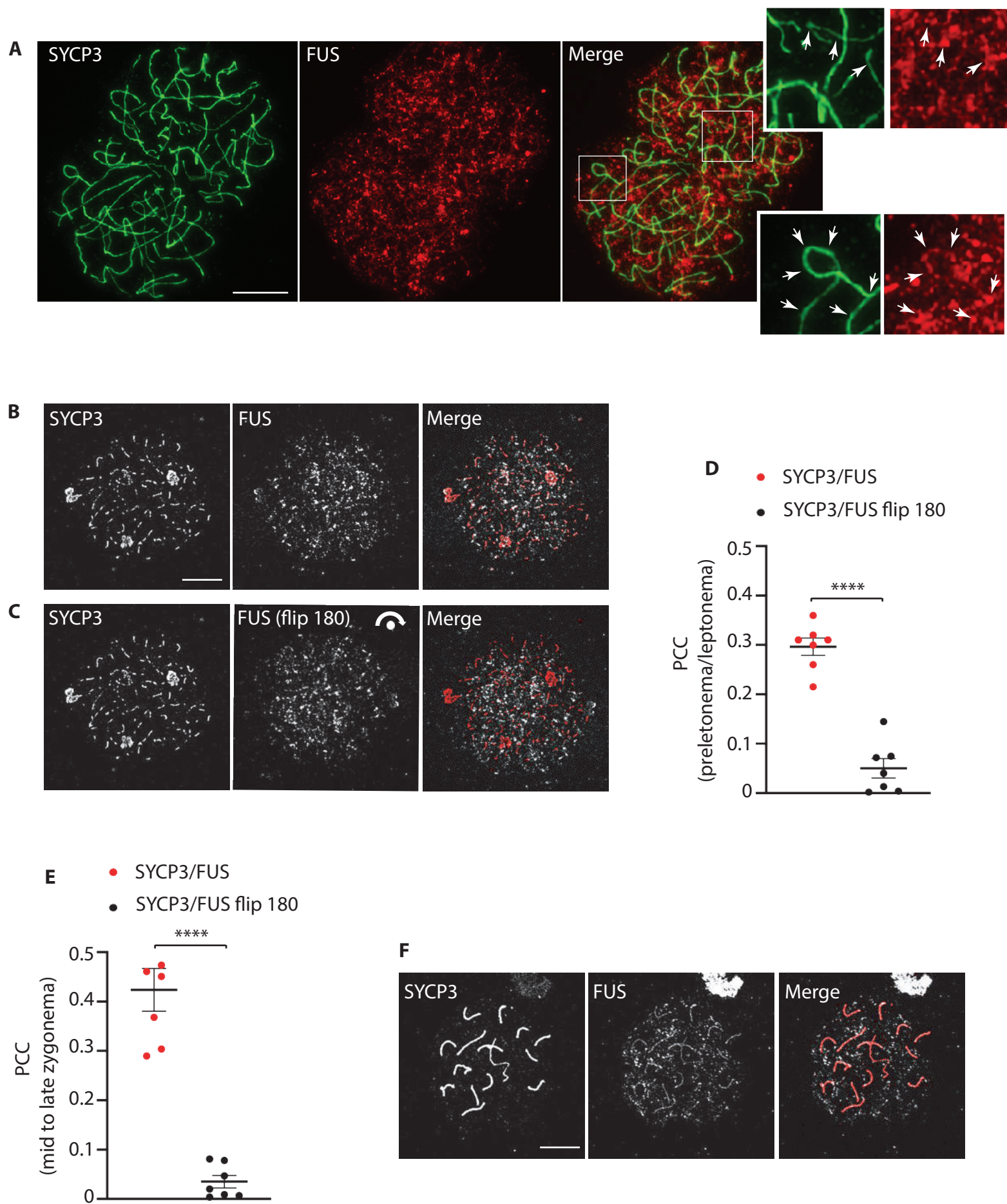

Fig. S1

Supplement: Supplementary file 2 — Supplementary file2 (PDF 1334 KB) [file 18_2023_4744_MOESM2_ESM.pdf]

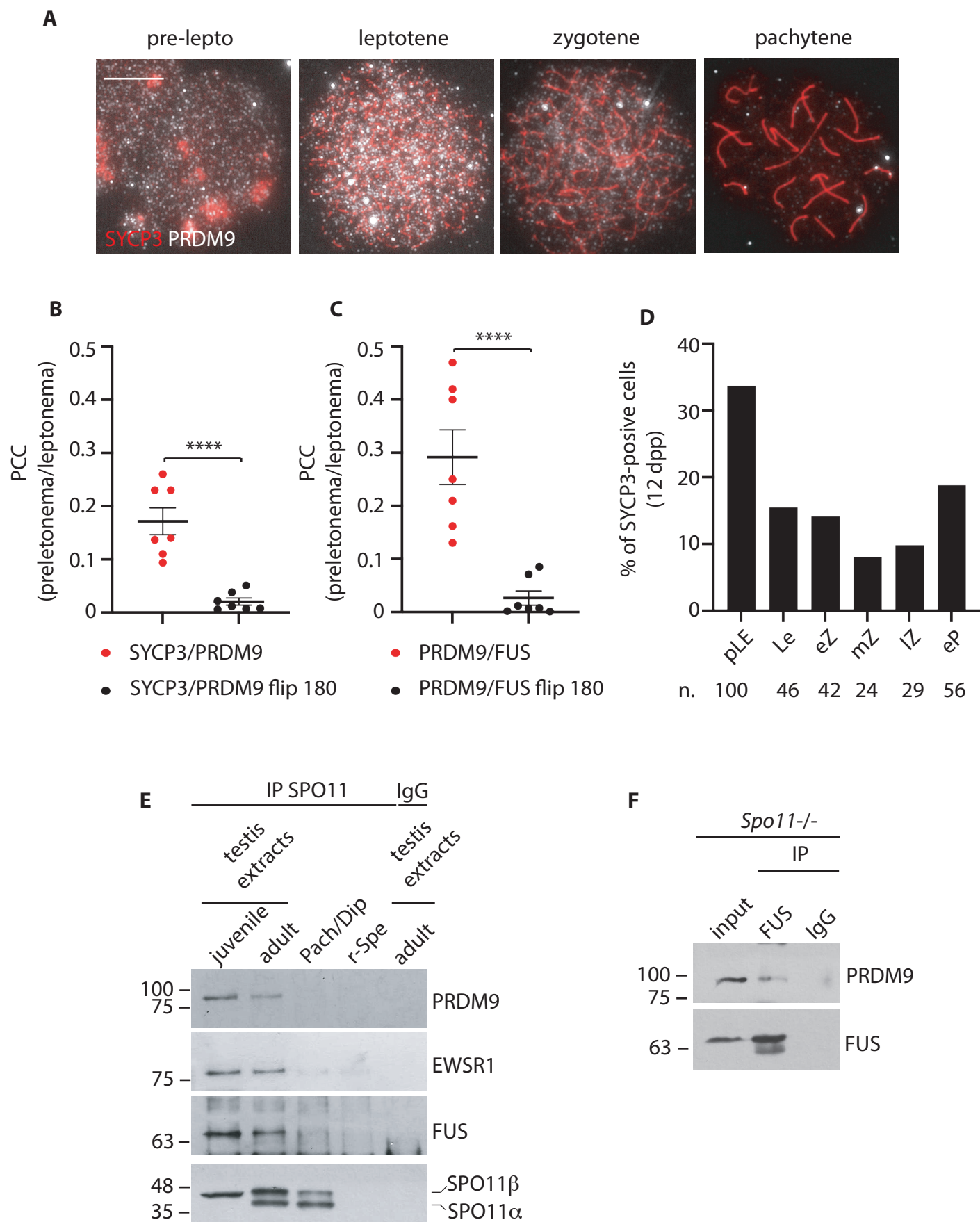

Fig. S2

Supplement: Supplementary file 3 — Supplementary file3 (PDF 16631 KB) [file 18_2023_4744_MOESM3_ESM.pdf]

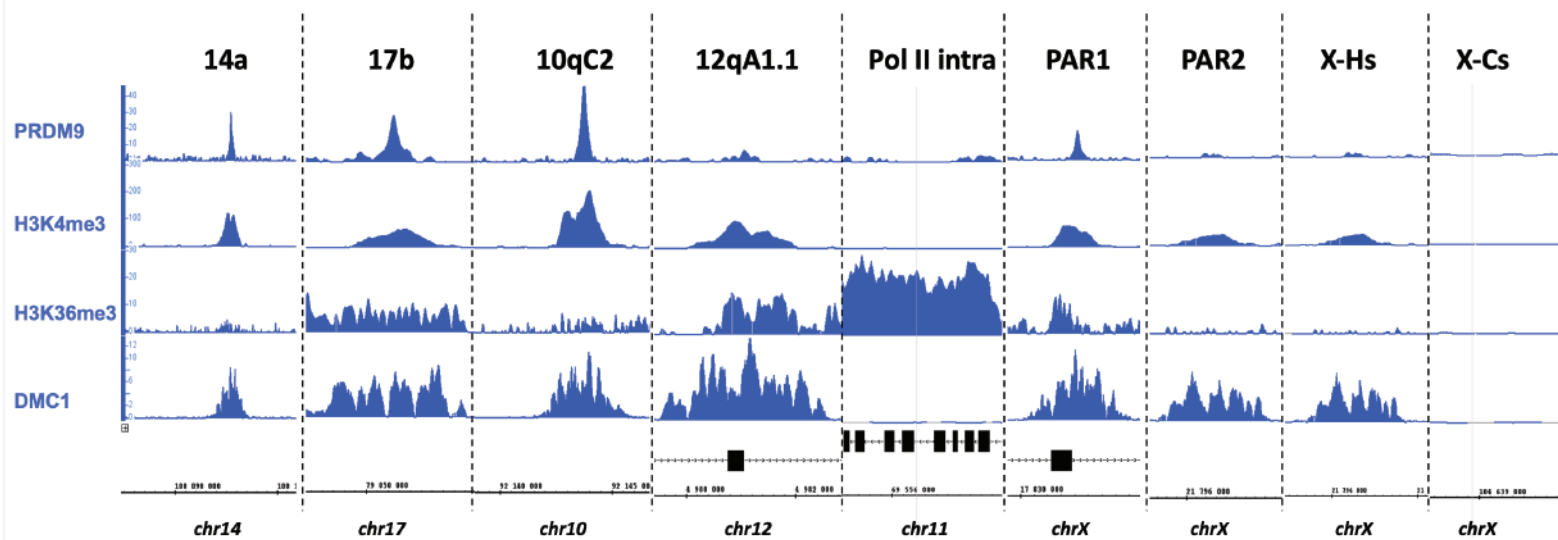

Fig. S3

Supplement: Supplementary file 4 — Supplementary file4 (PDF 85 KB) [file 18_2023_4744_MOESM4_ESM.pdf]
