# Supplementary material for: The RNA-binding protein FUS/TLS interacts with SPO11 and PRDM9 and localize at meiotic recombination hotspots
Source: Cell Mol Life Sci. 2023 Mar 26;80(4):107. doi: 10.1007/s00018-023-04744-5 (PMC10040399; doi:10.1007/s00018-023-04744-5)
Supplement: Supplementary file 5 — Supplementary file5 (DOCX 23 KB) [file 18_2023_4744_MOESM5_ESM.docx]

# Table S1A. Antibodies used for WB, IF, IP and ChIP: application, source and working dilution

| **Antibodies** | **Application** | **Species** | **Source** |
| --- | --- | --- | --- |
| H3K4me3 | ChIP | Rabbit | Merck Millipore 07473 |
| FUS (4H11) | IP  WB 1:1000  IF 1:300 | Mouse | Santa Cruz sc-47711 |
| SYCP3 | IF 1:300 | Rabbit | Novus Biologicals NB 300-231 |
| SYCP3 | WB 1:1000 | Guinea Pig | Bernard de Massy^1^ |
| SYCP3 | IF 1:300 | Rat | Mary Ann Handel^2^ |
| SPO11 | IP  WB 1:1000 | Mouse | Scott Keeney^3^ |
| IGg2A | IP | Mouse | Sigma-Aldrich M5409 |
| TUBULIN | WB 1:1000 | Mouse | Sigma-Aldrich T4026 |
| PRDM9 | WB 1:500  IF 1:100 | Rabbit | Corinne Grey^1^ |
| REC114 | WB 1:1000 | Rabbit | Attila Toth^4^ |
| CLATHRIN | WB 1:1000 | Mouse | BD Bioscience 610500 |
| EWSR1 | WB 1:1000 | Rabbit | Maria Paola  Paronetto^5^ |

WB: western blotting; IF: immunofluorescence; IP immunoprecipitation; ChIP: Chromatin immunoprecipitation

1. Diagouraga B, Clement JAJ, Duret L, Kadlec J, de Massy B, Baudat F. PRDM9 Methyltransferase Activity Is Essential for Meiotic DNA Double-Strand Break Formation at Its Binding Sites. Mol Cell. 2018;69(5):853-65 e6. Epub 2018/02/27. doi: 10.1016/j.molcel.2018.01.033. PubMed PMID: 29478809.
2. Eaker S, Pyle A, Cobb J, Handel MA. Evidence for meiotic spindle checkpoint from analysis of spermatocytes from Robertsonian-chromosome heterozygous mice. J Cell Sci. 2001;114(Pt 16):2953-65. Epub 2001/11/01. doi: 10.1242/jcs.114.16.2953. PubMed PMID: 11686299.
3. Neale MJ, Pan J, Keeney S. Endonucleolytic processing of covalent protein-linked DNA double-strand breaks. Nature. 2005;436(7053):1053-7. Epub 2005/08/19. doi: 10.1038/nature03872. PubMed PMID: 16107854; PubMed Central PMCID: PMCPMC1262668.
4. Stanzione M, Baumann M, Papanikos F, Dereli I, Lange J, Ramlal A, et al. Meiotic DNA break formation requires the unsynapsed chromosome axis-binding protein IHO1 (CCDC36) in mice. Nat Cell Biol. 2016;18(11):1208-

20. Epub 2016/10/28. doi: 10.1038/ncb3417. PubMed PMID: 27723721; PubMed Central PMCID: PMCPMC5089853.

5. Paronetto MP, Minana B, Valcarcel J. The Ewing sarcoma protein regulates DNA damage-induced alternative splicing. Mol Cell. 2011;43(3):353-68. Epub 2011/08/06. doi: 10.1016/j.molcel.2011.05.035. PubMed PMID: 21816343.

# Table S1B. Secondary antibodies used for WB, IF, IP: application, source, and working dilution

| **Secondary antibodies** | **Application** | **Source** |
| --- | --- | --- |
| Protein A (HRP) | WB 1:10.000 | BioLegend 689202 |
| Goat α Guinea Pig (IgG H+L) (HRP) | WB 1:10.000 | Novus Biologicals NB 7398 |
| Mouse  (m-IgGk BP-HRP) | WB 1:10.000 | Santa Cruz sc-516102 |
| Goat α Rabbit (IgG-HRP) | WB 1:10.000 | Santa Cruz sc-2054 |
| Alexa Fluor 568 Mouse | IF 1:400 | Life Technologies A11004 |
| Alexa Fluor 647 Rabbit | IF 1:400 | Life Technologies A21244 |
| Alexa Fluor 488 Rabbit | IF 1:400 | Life Technologies A11008 |
| Goat Anti-Mouse IgG 488 | IF 1:400 | SouthernBiotech 1031-30 |
| Alexa Fluor 594 Rat | IF 1:400 | Life Technologies A48264 |

WB: western blotting; IF: immunofluorescence

**Table S2. Primers used for ChIP qPCR**

| **Locus** | **Name** | **Sequence_F** | **Sequence_R** |
| --- | --- | --- | --- |
| **Chromosome 14** | **14a** | TCCTGGTAATTTTTCTTGCTTT | CCAATCCAATGTCCCTTACA |
| **Chromosome 17** | **17b** | CATGGACATGGAGACCTAACTG | TCAGTGGAAGCTCAGAAAATGA |
| **Chromosome 10** | **10qC2** | GCCAAGTAAGCTCTTCCTCC | TGACTTCTGCCTACCACTCA |
| **Chromosome**  **12** | **12qA1.1** | AACGTCCAGCCTAATTGTCC | GAATCAGGACCTCAGGCAAG |
| **Intragenic**  **region Polr2a** | **Pol II- Intra** | CTGTCTCCAGGTCATTGAGAAGG | GTTGATCTTGGAACCCTTGGCTCC |
| **Chromosome X** | **PAR1** | CTCCTGGTGATCGAGTCAGG | GGAAGAAAGTCTCAGGCCAT |
| **Chromosome X** | **PAR2** | ACAAGAGTGTCTATACTGCTCAGG | GATCTCCACACTAGCTGGCG |
| **Chromosome X** | **X-Hs** | GTTCATGCACTATATTGTGGTTATA | GACCTAAACACAAATACATGCACAC |
| **Chromosome X** | **X-Cs** | TGTTGACTTTGGCTGTGCAA | AGGACAACATGGCAGGAGTG |
